# Protective Effects of PACAP in Diabetic Complications: Retinopathy, Nephropathy and Neuropathy

**DOI:** 10.3390/ijms26199650

**Published:** 2025-10-03

**Authors:** Dora Reglodi, Andrea Tamas, Inez Bosnyak, Tamas Atlasz, Edina Szabo, Lina Li, Gabriella Horvath, Balazs Opper, Peter Kiss, Liliana Lucas, Grazia Maugeri, Agata Grazia D’Amico, Velia D’Agata, Eszter Fabian, Gyongyver Reman, Alexandra Vaczy

**Affiliations:** 1 HUN REN-PTE PACAP Research Group, Department of Anatomy, University of Pecs Medical School, 7624 Pecs, Hungaryattam@gamma.ttk.pte.hu (T.A.); edina.szabo@aok.pte.hu (E.S.); vaczyalexandra@gmail.com (A.V.); 2 Department of Sports Biology and Kinesiology, Faculty of Sciences, University of Pecs, 7624 Pecs, Hungary; 3 Section of Anatomy, Histology and Movement Sciences, Department of Biomedical and Biotechnological Sciences, University of Catania, 95123 Catania, Italy; graziamaugeri@unict.it (G.M.); vdagata@unict.it (V.D.); 4 Department of Drug Sciences, University of Catania, 95123 Catania, Italy

**Keywords:** diabetic nephropathy, diabetic retinopathy, diabetic neuropathy, diabetic keratopathy

## Abstract

Pituitary adenylate cyclase-activating polypeptide (PACAP) is a neuropeptide exerting, among others, strong trophic and protective effects. It plays a role in several physiological functions, including glucose homeostasis. The protective effects of PACAP are mainly mediated via its specific PAC1 receptor by stimulating anti-inflammatory, anti-apoptotic and antioxidant pathways. The aim of the present review is to summarize data on the protective effects of PACAP in the three major complications of diabetes, retinopathy, nephropathy and neuropathy, as well as some other complications. In type 1 and type 2 diabetic retinopathy models and in glucose-exposed cells of the eye, PACAP counteracted the degeneration of retinal layers and inhibited apoptosis and factors leading to abnormal vessel growth. In models of nephropathy, kidney morphology was better retained after PACAP administration, with decreased apoptosis and fibrosis. In diabetic neuropathy, PACAP protected against axonal–myelin lesions and less activation in pain processing centers. This neuropeptide has several other beneficial effects in diabetes-induced complications like altered vascular response, cognitive deficits and atherosclerosis. The promising therapeutic effects of PACAP in several pathological conditions have encouraged researchers to design PACAP-related drugs and to develop ways to enhance tissue delivery. These intentions are expected to result in overcoming the hurdles preventing PACAP from being introduced into therapeutic treatments, including diabetes-related conditions.

## 1. Introduction

Pituitary adenylate cyclase-activating polypeptide (PACAP) was discovered based on its cAMP stimulating effect in the hypophysis [1]. PACAP occurs in two biologically active forms, with 27 or 38 amino acid residues (PACAP27 and PACAP38, respectively), with the 38 form being dominant in mammals. PACAP is a conserved neuropeptide, acting on three G protein-coupled receptors (PAC1, VPAC1 and VPAC2), and it can also traverse cellular membranes to act directly on intracellular targets [1,2]. The diversity of the receptors, as well as the activity-dependent expression of the peptide and its receptors, can explain the diversity in functions [3,4,5]. In addition, PACAP can activate various signaling pathways in a cell- and tissue-specific manner and depending on activity and differentiation status [6,7]. It is therefore not surprising, similarly to many other neuropeptides, that PACAP plays a role in several physiological and pathological conditions. Among others, it is involved in central behavioral processes such as stress, anxiety and locomotor regulation [8,9,10,11]; in cognitive functions [12]; in thermogenesis [13]; in central and peripheral cardiovascular and respiratory regulation [14,15,16]; in bone and cartilage development and maintenance [17,18] and in migraine pathogenesis [6,19].

**Figure 1:**
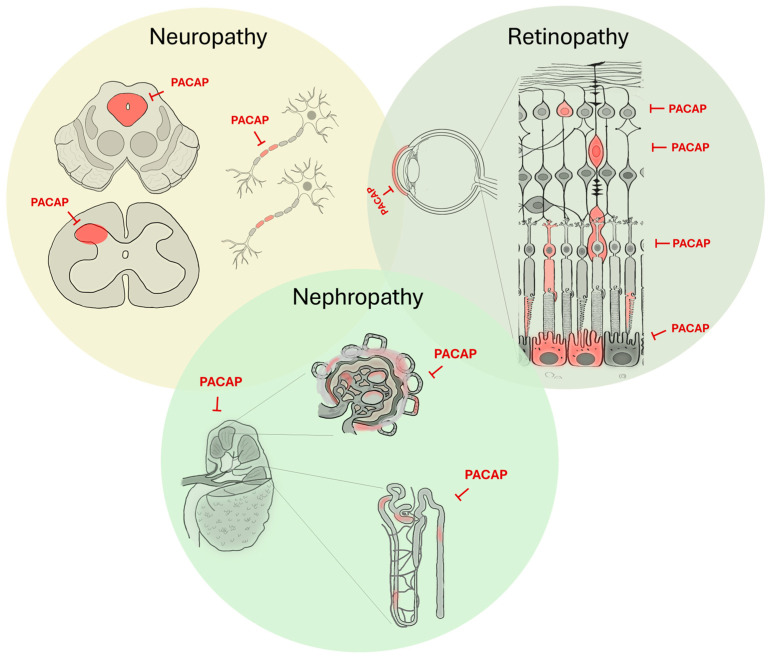
A graphical summary of the main findings regarding PACAP’s protective effects in major diabetic complications.

One of the most intensively studied actions of PACAP is its cellular protective effects exerted in different neuronal and non-neuronal cells [20,21,22]. This cytoprotective action is mediated mainly by the PAC1 receptor, involving anti-apoptotic, anti-inflammatory and antioxidant effects. In vitro, PACAP has been shown to protect against, for example, cardiomyocyte apoptosis, endothelial dysfunction, cerebellar granule cell death, bacterial infections and intestinal inflammations [23,24,25,26,27]. In vivo, this peptide is protective in animal models of neurodegenerative diseases, cardiomyopathy, colitis, ischemic lesions or amyloidosis [1,20,28,29,30]. Diabetes is associated with several macro- and microvascular complications, affecting most organs, especially the kidney, eye and peripheral nerves. The protective effects of PACAP against different in vitro and in vivo toxic agents and factors leading to degenerative changes have already drawn attention to PACAP as a protective agent in diabetic complications [31]. The aim of the present review is to summarize data on the protective effects of PACAP in the three major complications of diabetes, diabetic retinopathy, nephropathy and neuropathy, as well as some other complications [32]. The main findings are summarized in Figure 1 and Table 1.

## 2. PACAP in the Pancreas

The presence of PACAP and its receptors has been described in both the exocrine and endocrine pancreas [54,55,56,57]. PACAP is abundant in the Langerhans islets, and it is also involved in the nervous control of hormonal secretion. Intrapancreatic ganglia and sensory, parasympathetic and enteric fibers innervating the endocrine pancreas contain PACAP [56]. In humans, immunohistochemistry revealed that PACAP and the PAC1 receptor are present in the exocrine and endocrine parts of the pancreas [54,58]. PACAP immunoreactivity was weak in the acini and very strong in the islets of the normal pancreatic tissue, while it almost disappeared in ductal adenocarcinoma samples [54]. PAC1 receptor immunostaining was very strong in both the exocrine and endocrine parts and was found to be markedly weaker in tumor samples [54]. In insulinoma samples, strikingly weaker immunostaining was observed, while staining disappeared in the exocrine part in chronic pancreatitis [58].

PACAP is known to participate in the regulatory mechanisms of insulin and glucagon secretion and to be involved in glucose homeostasis in animals and humans [59,60,61,62,63]. Therefore, PACAP is considered as an insulinotropic peptide in the islets of Langerhans [64]. PACAP stimulated both insulin and glucagon secretion at high glucose concentrations [65,66,67], while at basal glucose concentrations, only glucagon was increased [66,68,69]. Daily injections of the antagonist PACAP6-27 for 14 days impaired glucose tolerance, insulin sensitivity and the glycemic response to feeding without altering glucagon, basal insulin, plasma glucose or lipid levels; feeding activity or body weight in ob/ob mice [70]. The acute administration of PACAP1-27 caused an elevation in blood glucose and insulin secretion, while acute PACAP6-27 could antagonize the insulinotropic action of PACAP1-27 without altering glucose levels [70]. On a long-term basis, PACAP exerted the transcriptional regulation of the insulin gene and of the genes of the glucose transporter and hexokinase in a rat insulinoma cell line [71]. Other studies found that PACAP could directly stimulate glucose output from hepatocytes [72,73]. PACAP also participates in the adrenergic counter-response to hypoglycemia, during which PACAP is released from the sympathetic splanchnic nerves innervating the adrenal medulla [74]. It is also known that PACAP participates in the regulation of glucose homeostasis not only at the peripheral level but centrally, in the ventromedial hypothalamus [75,76]. Furthermore, PACAP is an important component in the hypothalamic control of hepatic glucose production [77]. The insulinotropic effect of PACAP was significantly impaired in diabetic islets in spite of the increased cAMP response and unaltered co-localization pattern [78]. Similarly to PACAP, a cyclopeptide derived from the cyclization of PACAP(1-5) activating the PAC1 receptor could decrease plasma glucose levels, increase insulin levels and improve glucose tolerance in vivo [79]. A recombinant, slow-release PACAP-derived peptide could also alleviate diabetes by promoting both insulin secretion and action [80]. In a transplantation model, PACAP elevated insulin secretion in vivo [81]. The indirect effects of PACAP through the regulation of selenoprotein T have also been shown to be involved in the control of glucose homeostasis [82]. A recent study identified two viral peptides that are structurally similar to PACAP and implied that viral peptides may be linked to PACAP-stimulated glucose-dependent insulin secretion [83].

The overexpression of PACAP could inhibit hyperinsulinemia and islet hyperplasia in agouti yellow mice [84,85]. Transgenic mice overexpressing PACAP in pancreatic islets showed increased insulin levels, attenuated streptozotocin-induced plasma glucose and an age-dependent increase in islet cell mass [86]. PACAP administration could retard the onset of hyperglycemia by maintaining the beta cell mass in calmodulin-overexpressing diabetic mice [87]. Exogenous PACAP administration for 7 weeks prevented the development of hyperglycemia in Goto–Kakizai (GK) rats, emphasizing the peptide’s antidiabetic effects [88]. In turn, PACAP knockout (KO) or PAC1 receptor-deficient mice have metabolic disturbances. PACAP KO mice had unaltered serum insulin and glucose levels in the fed state, while increased insulin and decreased glucose levels were observed in fasting animals [89]. Another study found a 30% decrease in insulin levels, a reduction in glucose levels and a temperature-dependent increase in insulin tolerance [90]. Mice with virus-mediated PACAP deletion in the mediobasal hypothalamus rapidly gained weight and became hyperinsulinemic and hyperglycemic [91]. Another study, on the other hand, found hypoinsulinemia, reduced fat tissue mass, and lower body weight and food intake in whole-body PACAP KO mice [92]. PACAP-deficient mice also displayed other metabolic disturbances with increases in lipid parameters [89,93]. PAC1 receptor-deficient mice had 50% reduced insulin response to raising glucose concentrations in the perfused pancreas [94] and disturbed lipid metabolism [95]. Several other metabolic effects of PACAP can be related to diabetes, like its anorectic and anti-obesity effects, although no direct connection has been established yet [96,97].

A recent study found that PACAP could ameliorate insulin resistance, characterized by increased glycogen synthesis and the suppression of gluconeogenesis, in an insulin-resistant cell model and in mice fed with a high-fat diet [98]. In obese mice, the authors found that PACAP treatment reduced body weight, blood glucose levels and food intake [98]. Several other studies have described PACAP’s participation in the regulation of food intake, nutrition, thermoregulation, energy balance, metabolic functions and glucose tolerance [99,100,101,102,103]. It could attenuate hepatic lipid accumulation and inhibit lipogenesis in high-fat-diet-fed mice [100]. PACAP plays a role in various other metabolic processes in addition to the above described effects, which can also contribute to the factors in diabetes generation and complications [104,105,106,107,108]. As PACAP is involved in the regulation of insulin secretion and the PACAP gene is located on chromosome 18p11, where a genome-wide scan revealed an association between type 2 diabetes and obesity, it was presumed that PACAP gene variations could also be one of the factors accounting for the development of diabetes. A Scandinavian population scan found, however, that the variants of the Adcyap1 gene may not be a major influencing factor in the Swedish and Finnish populations [109].

Studies have shown that the general cytoprotective actions of PACAP are also present in pancreatic islets. Vagal PACAP can function synergistically with cholinergic signaling to increase beta cell proliferation via the forkhead box cell cycle transcriptional factor [110]. The protective effects of endogenous PACAP were shown in PACAP-null mice islets, which displayed increased sensitivity to gluco- and lipotoxicity [111]. Treatment with high glucose or palmitate not only caused impaired calcium and insulin response but also led to the impaired mRNA expression of uncoupling protein 2 [111]. The protective effects of a PACAP38 analog were also proven in rat insulinoma cells against STZ-toxicity. The chronic administration of this analog protected islets in neonatally STZ-injected rats, followed by an improvement in glycemic control [112]. Protection was also shown in the rat islet cell line RINm5F treated with conditioned medium from PACAP-preconditioned mesenchymal stromal cells exposed to high glucose and streptozotocin [113]. In these cells, viability was increased, mitochondrial membrane potential was restored and oxidative stress-induced apoptosis was reduced. Oxidative stress markers were also altered: radical oxygen species were reduced, while the antioxidant activity of glutathione peroxidase was increased. The functional recovery of the cells was proven by increased insulin levels [113]. PACAP could also improve beta cell viability and survival during culture, with increased insulin secretion [81]. All these results imply the potential therapeutic role of PACAP along with VIP in diabetes [31,114].

## 3. PACAP in Diabetic Retinopathy—In Vivo Studies

Diabetic retinopathy is one of the most feared complications of diabetes that affects a high percentage of patients, leading to severe visual impairment. The presence of PACAP and its PAC1 receptor has been mapped in rodent and human retinas [115,116,117,118,119,120,121]. The protective effects of PACAP have been shown in several models of retinopathies, including optic nerve transection, glaucoma, ischemic retinopathy, excitotoxity-induced injury, UV light-induced retinopathy and retinopathy of prematurity [116,122,123]. Endogenous PACAP is also protective in the retina, as PACAP KO mice displayed early retinal aging [124] and increased susceptibility to retinal ischemia-induced degeneration [125] or retinopathy of prematurity [126]. The distribution of PACAP and the PAC1 receptor in the human eye was found to be very similar or identical to that in rodents, implying that the protective effects observed in rats and mice could be translated to humans [115]. Diabetes affected the endogenous expression of PACAP, as mRNA for PACAP and its receptors were reduced in diabetic rat retinas [41].

Regarding diabetes, several studies have proven the potency of PACAP in protecting against diabetes-induced retinal damage [127]. The first in vivo study revealed that intravitreal PACAP treatment in type 1 diabetic rats, induced by a one-time higher dose of streptozotocin, led to less severe damage in the retina [34]. Ganglion cell loss and Müller glial cell overactivation were reduced by PACAP. This latter effect could also be a direct effect on Müller glial cells, as these cells are direct target cells for PACAP’s action [128]. The terminals of cone photoreceptor outer segments were damaged in diabetic rats, which was not observed after PACAP treatment. Furthermore, in PACAP-treated animals, the dopaminergic amacrine cell structure was retained [34]. These results prompted further research, as PACAP turned out to be a promising peptide in treating diabetic retinopathy [129,130]. Since PACAP did not change body weight or blood glucose levels, molecular mechanisms targeting cell death or other pathways must be responsible for the observed protective effects. In a follow-up study, more insight into the molecular mechanism was gained [35]. Intraocular PACAP could increase the expression of anti-apoptotic p-Akt, p-ERK2, PKC and Bcl-2 in diabetic rats. In contrast, the treatment downregulated pro-apoptotic phospho-p38MAPK and caspase-3, -8 and -12. This led to a significant decrease in the number of apoptotic cells in all nuclear layers of the retina. The degeneration of the dopaminergic amacrine cells is among the first signs of diabetic retinopathy. The apoptosis of these cells was increased in diabetic samples, while PACAP treatment could decrease the death of these cells. These results were in accordance with another study, where a single intravitreal injection of PACAP could counteract the diabetes-induced decrease in Bcl-2 and p53 transcript levels three weeks after the onset of diabetes [41]. This study, in addition, revealed that an acute transient increase in retinal PACAP, VPAC1 and VPAC2 receptor expression is followed by a significant decrease three weeks later [41]. The anti-apoptotic effects of PACAP are well established in several cell types under different pathological conditions, including cerebellar granule cells, cortical neurons, astrocytes, neuroblastoma cells, intestinal and kidney tubular cells, hepatocytes, cardiomyocytes and PC12 cells [1,20,131,132,133,134,135,136,137,138,139,140,141]. These results in a rat model of diabetic retinopathy showed that the retinoprotective effects are, at least partly, achieved by the anti-apoptotic effects of PACAP in the retina [35]. In a similar experimental setup, PACAP modulated hypoxic processes, as marked changes in hypoxia-inducible factor expression were found [37]. Diabetes induced an increased expression of HIF-1alpha and HIF-2alpha, along with a marked downregulation of HIF-3alpha. All these changes were counteracted by intraocular PACAP injections [37]. HIF-1alpha and HIF-2alpha promote growth factors that lead to neovascularization typical for diabetic retinopathy, while HIF-3alpha, a negative modulator of the other HIFs, inhibits these processes. The changes induced by PACAP confirm that the peptide is able to counteract the molecular mechanisms responsible for pathological vessel formation [37]. PACAP was also able to induce the retinal expression of the protective peptide ADNP (activity-dependent neuroprotective peptide) and reduce VEGF, VEGF receptor and interleukin-1 beta expression in diabetic rats, thereby implying the involvement of other induced factors in PACAP’s protective effects [38,39,142].

Electron microscopical examination revealed that in the same diabetic retinopathy model, pigment epithelial cells, ribbon synapses and other synaptic profiles suffered from alterations [36]. Horseshoe-shaped ribbon synapses were more retained in PACAP-treated retinas, and the number of cone bipolar and ganglion cells was restored. The outer segments of the photoreceptors, pigment epithelial cells and the outer limiting membrane suffered severe degeneration in diabetic retinas, which was prevented by PACAP treatment. The reduction in GLUT1 immunoreactivity was also prevented by PACAP treatment, and the increased expression of RAGE mRNA was reduced. PACAP could counteract the diabetes-induced reduction in the expression of the PAC1, VPAC1 and VPAC2 receptors. These results indicate that PACAP can help to maintain the integrity of the vertical retinal pathway [36].

Similar protective effects were observed in spontaneously hypertensive rats with induced diabetic retinopathy [40]. It was found that PACAP alone had some protective effects, but better results could be obtained when PACAP was combined with the PARP inhibitor olaparib. Double treatment could restore the decrease in the thickness of retinal layers and the ganglion cell number and could partially restore the altered staining patterns of the calcium-binding proteins, calbindin and parvalbumin [40].

A recent study has found similar protective effects in a rat model of type 2 diabetes [42]. Type 2 diabetes was modeled in rats with low-dose streptozotocin and a high-fat diet for 16 weeks. Twice-daily treatment with PACAP eye drops did not alter body weight or triglyceride levels. Blood glucose levels were slightly, though not significantly, lower in the PACAP-treated group. However, marked differences were observed in the visual function tested with electroretinography: the striking reductions in a- and b-waves and oscillatory potential were ameliorated by PACAP treatment in diabetic animals. The total retinal thickness, measured by optic coherence tomography, was well preserved in the PACAP-treated animals, as shown by the thickness of the sublayers, such as INL, IPL, ONL and photoreceptor outer segments. The microvascular structure was also protected: PACAP could counteract the vessel density-decreasing effect of diabetes in the periphery and edge regions of the retina, the decrease in pericyte survival and the increase in the number of acellular capillaries [42]. These results show that PACAP is protective not only in type 1 but also in type 2 diabetic retinopathy. Naturally, the translational limitation of animal studies with type 2 diabetes is that the time-course is much shorter in animals for the development of the disease than in humans, even though the 16-week-period in the mentioned model can be considered as a chronic treatment. Furthermore, these results also prove the effectiveness of PACAP treatment in the form of eye drops, not only via invasive intravitreal administration. The passage of PACAP through the ocular barriers has been tested previously, and it could be proven that PACAP, through eye drops on the cornea, reaches the retina and exerts retinoprotective effects in models of retinal ischemia and glaucoma [143,144].

## 4. In Vitro Protective Effects of PACAP in Retinal Cells Exposed to Hyperglycemia or Other Insults

PACAP’s retinoprotective effects were also studied in vitro in different cells of the retina and in retinal explants. In retinal explant preparations exposed to high glucose to mimic diabetic conditions, 100 nM PACAP could counteract the elevated caspase-3 and VEGF levels [47]. Pigment epithelial cells are connected to each other by tight junctions forming the outer retinal barrier and play a role in photoreceptor functions and regeneration. Their dysfunction lies in the background of several ocular complications, including diabetic retinopathy [50,145,146]. Not only hyperglycemia itself but also hypoxia, oxidative and hyperosmotic stress are additional aggravating factors in diabetic retinopathy [146,147,148]. PACAP has been shown to protect pigment epithelial cells exposed to various stressors, including hypoxia, hyperosmotic stress and oxidative stress [122,147]. In accordance with other findings, PACAP could reduce the expression of HIF-1alpha in pigment epithelial cells, reduce apoptosis and decrease the expression of cytochrome-c and p53, while it could increase the levels of antioxidant superoxide dismutase 2, thioredoxin and paraoxonase2 in a human pigment epithelial cell culture (ARPE19 cells) [146]. Another study found that PACAP ameliorated the pigment epithelial cell junctional disruption measured by the decrease in the transmembrane protein occludin and the plaque protein ZO-1 in cells exposed to osmotic (sucrose) or oxidative stress (hydrogen peroxide) [145]. PACAP co-administration could also moderate the cytoskeletal disorganization analyzed by atomic force microscopy in cells exposed to osmotic stress. This led to the restoration of the worsened elasticity of the cells. PACAP treatment could also mitigate the upregulation of angiogenic factors, such as VEGF, angiogenin and endothelin-1, in cells exposed to hyperosmotic or oxidative stress. All these effects could play a role in the effect of PACAP increasing cell survival [145]. Similar results were obtained in the same cells exposed to a hyperglycemic/hypoxic insult: the decrease in the junctional proteins ZO-1 and occludin was ameliorated, and PACAP increased transepithelial electrical resistance, indicating the preservation of the outer retinal barrier [38]. Furthermore, PACAP treatment could decrease choriocapillaris neovascularization, increase ADNP expression and decrease VEGF expression [38]. In another study with ARPE19 cells, reduced HIF-1alpha and increased HIF-3alpha levels were detected upon PACAP treatment [49]. PACAP could also decrease the hyperglycemia/hypoxia-exposed increase in VEGF and receptor expression and decrease the elevated apoptotic p53 expression level [49]. Under the same conditions, these protective effects were shown to be mediated through anti-apoptotic actions, involving the PI3K/akt and MAPK/ERK pathways [48]. In pigment epithelial cells exposed to a combination of high glucose and inflammation by interleukin-1beta, PACAP administration could reverse the increased permeability and the reduction in the junctional proteins claudin-1 and ZO-1 [48]. All these experiments using human pigment epithelial cells show that PACAP can counteract the changes induced by high glucose and other harmful effects also accompanying diabetic conditions, such as hyperosmolarity and inflammation.

## 5. PACAP and Diabetic Keratopathy

Diabetic keratopathy (DK) represents the major complication of the cornea characterizing diabetes-affected patients [149]. DK is associated with the hyperglycemic state leading to persistent corneal epithelial defects, superficial punctate keratopathy, alteration of the normal wound healing mechanism with delayed epithelial regeneration, recurrent corneal ulcers and reduced corneal sensitivity [150]. The expression of PACAP and its receptors in the cornea has been well-documented, providing a foundation for its therapeutic potential [151]. Studies on rabbit and human corneal tissue have identified PACAP-positive cells primarily in the basal layer of the epithelium [115]. The expression of the peptide and its receptors was also detected in the corneal endothelial layer and in the stroma [152,153,154]. In addition, PACAP immunoreactivity has been detected in the nerve terminals that extend through the stroma, with branches reaching into the epithelium [155]. A lot of evidence has shown the protective role of PACAP in the cornea. In an experimental model of laser-assisted in situ keratomileusis (LASIK) surgery, treatment with PACAP promoted the recovery of corneal sensitivity following the creation of a corneal flap [156]. Moreover, the peptide, alone or in combination with the N-terminal agrin (NtA) domain, significantly induced the repair of mechanically injured corneal epithelial cells [157]. Interestingly, in a mouse model of corneal epithelial damage, a PACAP27-derived mutant peptide (MPAPO) accelerated wound closure after 36 h. Histological analysis confirmed a restored corneal structure with an intact epithelial layer 48 h post-administration. Furthermore, MPAPO promoted lacrimal secretion and axonal regeneration, with innervation patterns comparable to control groups. PACAP itself also stimulates lacrimal secretion and protects against dry eye symptoms [158]. The underlying mechanism involved the activation of the cAMP/PKA signaling pathway, which, in turn, enhanced cell proliferation in H_2_O_2_-injured corneal epithelial cells [159,160].

The protective effects of PACAP were also observed in the corneal endothelium. In human corneal endothelial cells (HCECs), isolated from the peripheral corneal–scleral tissue of a donor, PACAP treatment improved cell viability, maintained barrier integrity and promoted wound repair in growth factor-deprived monolayers [152,154]. The molecular mechanism behind this trophic effect is linked to the phosphorylation of the epidermal growth factor receptor (EGFR), leading to the activation of the MAPK/ERK1/2 signaling pathway [152]. The ability of PACAP to induce EGFR transactivation is a well-established phenomenon, observed in various cell types and models, including neurons and diabetic retinopathy models [161,162,163,164]. Furthermore, PACAP protected HCECs from UV-B radiation damage by promoting the formation of new tight junctions, which are essential for maintaining barrier integrity and proper fluid transport [153].

In the diabetic cornea, the expression levels of PACAP, PAC1R and EGFR were compromised. This condition could be responsible for the delayed epithelial wound healing observed in DK [52]. In rabbit corneal epithelial cells grown in high glucose to mimic a diabetic microenvironment, treatment with the peptide increased cell viability and promoted corneal epithelial wound healing via EGFR/ERK signaling pathway activation [52].

In an effort to better understand these mechanisms in vitro, studies have utilized the air–liquid interface (ALI) cornea model, which closely mimics the corneal epithelial barrier in a controlled environment. Experiments on this model have demonstrated that PACAP treatment significantly accelerated epithelial wound closure, restored tight junction protein expression (e.g., ZO-1) and reduced the expression of pro-inflammatory cytokines, further solidifying its role in maintaining corneal homeostasis [53]. The mechanism through which PACAP appears to counteract the inflammatory event triggered by high glucose levels is through the inhibition of the NF-kB signaling pathway, which is known to play a central role in the inflammatory response [165]. A recent study has confirmed the protective effects of PACAP in high-glucose-exposed human corneal epithelial cells [51]. PACAP was able to restore the glucose-induced impairment of cell proliferation capacity, autophagy and migration. PACAP also counteracted the high-glucose-induced decreased levels of Beclin1, LC3B, Ki-67 and Bcl-2 mRNA and the p-AMPK, p-ERK and Bcl-2 proteins [51]. In conclusion, by activating its specific receptors, PACAP enhances cell survival, decreases apoptosis, promotes epithelial proliferation and migration and reduces oxidative stress, thereby counteracting the pathological changes induced by the hyperglycemic environment. Its ability to regulate tight junction proteins and maintain epithelial barrier function is also critical for corneal integrity. These multifaceted actions highlight the potential of PACAP to address the core pathological features of diabetic keratopathy, offering a novel and comprehensive approach for the treatment of this debilitating ocular complication.

## 6. PACAP in Diabetic Neuropathy

Diabetic polyneuropathy is another main microvascular complication of diabetes [166]. Hyperglycemia and a reduction in oxygen delivery in the vessels of peripheral nerves lead to increased inflammation and increased oxidative stress resulting in endothelial dysfunction and hyperplasia, Schwann cell degeneration and dysfunction, demyelination, and a lower energy status of the nerves, as well as axonal degeneration [167,168]. Adenylyl cyclases are recognized therapeutic targets in neuroregeneration, as adenylate cyclase activity increases after nerve injury, and the cascade of events initiated by this activation is critical in axonal growth, growth cone dynamics and regeneration [169,170]. Soon after its discovery, it became evident that PACAP possesses neurotrophic and neuroprotective effects. Dozens of studies have described the involvement of PACAP in the development of the central and peripheral nervous system [171,172]. Thanks to all these observations, PACAP is now an accepted neurotrophic factor [5]. Trophic factors are known to be upregulated after injuries to promote regenerative processes [172], and PACAP, along with its receptors, is upregulated following peripheral nerve injury of different types [169,173,174,175]. PACAP has been proven to induce axonal growth [176,177] and to direct axonal growth cone elongation [178]. In the peripheral nervous system, PACAP could enhance nerve regeneration [179]. This effect is also present endogenously, as mice lacking endogenous PACAP showed significantly delayed axonal regeneration in a facial nerve injury model, accompanied by increased inflammatory cytokine levels of TNF-alpha, interleukin-6 and interferon gamma and decreased anti-inflammatory interleukin-4 levels [180].

Schwann cells are crucial in peripheral nerve regeneration; they are able to respond rapidly following injury to redifferentiate into a regeneration-supporting phenotype [181]. Schwann cells release several neurotrophic factors, such as NGF, BDNF, GDNF, VEGF and NT3. This list can be completed by PACAP, as Schwann cells have been shown to release PACAP [181]. In turn, it is now well established that PACAP influences Schwann cell functions in several ways [181,182]. PACAP receptors are highly expressed in Schwann cells and macrophages, and PACAP is able to promote myelin gene expression and inhibit pro-inflammatory cytokines (TNF-alpha, IL6, IL1alpha and beta, MCP1) [175]. Interestingly, the effect on pro-inflammatory cytokines could not be shown on unstimulated Schwann cells, only in inflammation-stimulated ones, and this effect was VPAC receptor-mediated [175]. The stimulatory effects of PACAP on anti-inflammatory cytokines (IL-4, 10, 13) and its inhibitory effects on pro-inflammatory cytokines (TNF-alpha, IL-6, IL1alpha and beta, MCP-1) were shown in sciatic nerve explants [175]. These results show that PACAP plays an important role in the distal nerve stump following injury to promote myelination and regulate inflammation [175]. PACAP has been proposed to play distinct regulatory roles in inflammatory processes in the early and later phases of neuronal regeneration [181]. PACAP promoted Schwann cell redifferentiation, and it could directly stimulate myelination by inducing the expression of major protein components of myelin, such as MBP, MAG and MPZ. Tissue plasminogen activator expression was also induced, which is necessary for the removal of debris after injury by degrading the extracellular matrix [181,182,183]. Furthermore, the general cytoprotective effects of PACAP [131] can also be observed in Schwann cells: PACAP stimulated the survival of Schwann cells in serum deprivation and stimulated the expression of other trophic factors, such as BDNF [181,182,184,185]. Conversely, regenerative processes influence the PACAP/PACAP receptor system, as has been shown in a Schwann cell line upon inflammatory challenge, where a subset of dysregulated miRNAs were identified to elicit a regulatory role in the PACAP/VIP system [186].

A recent study employing single-cell RNA sequencing in a model of peripheral nerve crush injury revealed that the PACAP gene Adcyap1 was among the key mediator genes in neuropathic pain and regeneration [187]. The repeated intrathecal administration of PACAP38 attenuated pain and promoted axonal regeneration. In contrast, gene silencing (Adcyap1 siRNA) or the administration of the PACAP antagonist PACAP6-38 led to mechanical hyperalgesia and delayed axonal regeneration [187]. PACAP could alleviate pain in the acute phase but not in the chronic phase. This study also confirmed previous findings showing that PACAP38 promoted axonal outgrowth, while PACAP6-38 inhibited it in a dorsal root ganglion neuronal culture [187]. These findings strengthen the potential of PACAP in axonal regeneration and in pain alleviation. The neurite outgrowth-promoting effects of PACAP were also shown in mouse dorsal root ganglia cultures, where PACAP and DPPIV inhibitors induced a strong elongating effect of nerve processes [188]. A very important human study further highlighted the translational potency of the nerve regeneration-promoting effect of PACAP. The skin transcriptional profile was analyzed in 60 patients undergoing carpal tunnel surgery [189]. The authors identified 31 genes that were differentially expressed after decompression. Among these, the PACAP gene (Adcyap1) was the main upregulated gene and was linked to intradermal nerve fiber regeneration [189]. They also confirmed the axonal growth-promoting effects of PACAP, as the peptide dose-dependently stimulated axonal outgrowth in human-induced pluripotent stem cell-derived sensory neurons [181,189]. A recent human study found that serum PACAP levels are increased in patients with polyneuropathy with neuropathic pain with or without diabetes, but whether it contributes to the pain experienced by these patients or whether it is a response to nerve injury cannot be concluded from these results [190]. All these experimental data provide evidence for the sensitive responses of PACAP upon nerve injuries and the nerve regeneration-promoting effect of PACAP.

In a rat model of diabetic neuropathy, rats were treated with streptozotocin, and body weight and blood glucose levels were monitored for 8 weeks. Animals received intraperitoneal injections of PACAP38 (20 µg/100 µL) every second day for 8 weeks. An electron microscopical analysis of the sciatic nerve preparations revealed significant nerve damage in diabetic animals, which was markedly attenuated in PACAP-treated rats [33]. The percentage of axon–myelin separation was significantly increased in diabetic rats, while it was attenuated to half in PACAP-treated samples, only slightly higher than that in normal controls. A marked elevation was found in the number of mitochondria due to mitochondrial fission, which was also attenuated by PACAP. Unmyelinated fiber atrophy was shown by the decrease in the unmyelinated fiber area in the diabetic rats. A smaller damaged area was observed after PACAP treatment. Furthermore, electron microscopy revealed basement membrane thickening in endoneurial capillaries, a damage sign that was not seen at all in PACAP-treated rats. In the same study, functional analysis was also conducted. Pressure and touch sensitivity increased to a lower extent in the PACAP-treated rats. Pain processing is known to be altered in diabetes, and chronic conditions lead to increased FosB expression in pain processing centers, such as the periaqueductal gray matter and dorsal horn of the spinal cord. In PACAP-treated animals, the marked increase in FosB expression could not be observed in either the PAG or I-II-III laminae of L4-5 dorsal horn segments, indicating that in PACAP-treated animals, the chronic activation of central pain processing pathways is less prominent [33]. In this experiment, similarly to others with the same model, body weight was severely decreased along with the elevated blood glucose levels, but neither body weight nor blood glucose levels were changed after PACAP treatment. This shows that the protective effects of PACAP in this model were not due to attenuating hyperglycemia but to the protective effects of PACAP on peripheral nerves.

## 7. PACAP in Diabetic Nephropathy

The protective effects of PACAP in the kidney have been extensively studied in several rodent models and also in humans. PACAP has been proven to be protective in kidney ischemia, myeloma kidney injury, cisplatin-induced renal failure and gentamicin-induced nephropathy [191,192]. The protective mechanisms are also present endogenously, as mice lacking the endogenous peptide were more susceptible to ischemic kidney injury [193]. In addition, very severe amyloidosis was present in the renal corpuscles in these mice already starting at early ages and leading to functional deterioration, as shown by increased creatinine levels [93]. Both complete lack and partial lack lead to similar age-related changes in the kidneys of heterozygous knockout mice [194].

Diabetic nephropathy (DN) is a major complication of type 1 and type 2 diabetes; hence it is the leading cause of chronic kidney disease [195]. PACAP treatment was shown to act against inflammatory processes in an in vitro model of diabetic nephropathy [196]. Due to the crucial role of inflammation in the pathogenesis of DN, mouse podocytes were treated with lipopolysaccharide (LPS). PACAP, acting on VPAC1 receptors, was able to downregulate the pro-inflammatory cytokines monocyte chemoattractant protein-1 (MCP-1) and interleukin-6 (IL-6) induced by LPS exposure. In the molecular background, more signaling components and pathways were proven to contribute. Effects after LPS exposure were directed through the cAMP/PKA signaling pathway, changing ERK phosphorylation and NFκB transnuclear localization [196].

Under in vivo circumstances, intraperitoneal PACAP administration was shown to alleviate the morphological damage in a rat model of diabetic nephropathy [45]. Streptozotocin-induced diabetes led to tubular damage, glycogen deposition and glomerular damage, which were suppressed by intraperitoneal injections of PACAP. Besides diminishing histological damage, PACAP was able to ameliorate the streptozotocin-induced activation of certain cytokines, such as CINC-1, TIMP-1, LIX, MIG and s-ICAM [45]. The molecular regulation of PACAP’s effect against streptozotocin-induced diabetes included the downregulation of pro-apoptotic phospho-p38 MAPK and cleaved caspase-3. In addition, it also decreased the p60 subunit of NFκB. Anti-apoptotic factors, ERK1/2 and pAkt, displayed a slight increase, which was significantly strengthened by PACAP. PACAP was also shown to mitigate the expression of fibrotic markers, collagen IV and TGF-beta1 [46]. Antioxidant glutathione expression was increased by PACAP. Electron microscopical examination revealed the segmental thickening of the basement membrane in diabetic glomeruli, while PACAP-treated animals did not display this focal thickening. In addition, no podocyte foot process flattening or broadening was observed in PACAP-treated animals [46]. PACAP’s renoprotective action against streptozotocin-induced damage could also be detected when it was administered intravenously [43,44]. Both histological changes (glomerular enlargement, tubular vacuolization) and the activation of TNF-alpha and TGF-beta1 were significantly decreased by PACAP administration [43,44]. Interestingly, in this study, hyperglycemia and body weight loss were also attenuated after PACAP treatment along with decreased proteinuria and polyuria [44].

## 8. Other Diabetic Complications

PACAP has been shown to alleviate further diabetic complications. Hyperglycemia-induced vascular dysfunction is a common complication of diabetes, and it lies in the background of several organ dysfunctions [197,198]. A 1 h hyperglycemic episode can already lead to endothelial dysfunction, which was alleviated by PACAP administration [197]. PACAP restored the reduced endothelium-dependent relaxation induced by acetylcholine and improved the endothelium-independent sodium-nitroprusside-induced relaxation of the carotid arteries. As a positive control, a combination of superoxide dismutase and catalase was used, and the effects of PACAP were comparable to those of these scavengers in spite of PACAP lacking direct scavenging activity [197]. An angiogenesis array revealed that hyperglycemia induced the expression of fibroblast growth factorbasic (FGF basic), matrix metalloproteinase 9 and the nephroblastoma overexpressed gene. This alteration was reversed by PACAP administration [197]. These results show that PACAP not only protects hyperglycemia-exposed vessels due to its vasodilator capacity but also protects endothelial cells and other components of the vessel against toxicity [6,199,200,201]. Similar protective effects were found in vessels exposed to high lipid concentrations [202], where PACAP counteracted endothelial and smooth muscle cell injury, increased the production of anti-atherosclerotic substances, reduced the production of lipid peroxide and inhibited the proliferation of the smooth muscle cells [202]. Similarly, the PAC1 agonist maxadilan reduced the occurrence of atherosclerotic plaques and lumen stenosis in apolipoprotein E-deficient mice fed with a cholesterol-enriched diet [203]. Maxadilan treatment also reduced the TNF-alpha-immunoreactive areas in plaques and could reduce the caspase-3 immunoreactivity in the tunica media, providing atheroprotective effects [203]. This effect was confirmed in PACAP-deficient mice, where aggravated atherosclerosis was observed [204]. As hyperlipidemia is a common feature of diabetes, these findings are also important from the diabetes point-of-view. High glucose concentrations can induce abnormal endothelial cell proliferation, which was shown to correlate with changes in PAC1 receptor expression and, to a lesser extent, with VPAC2 expression. This increase in cell viability was attenuated by PACAP treatment in murine microvascular endothelial cells in vitro [205], further supporting the protective effects of PACAP in hyperglycemia-induced abnormal vessel responses.

A recent study has shown that PACAP could also be involved in diabetes-associated cognitive impairment [206]. The downregulation of the angiotensin-(1-7)/Mas receptor system has been implicated in the pathomechanism of diabetes-associated cognitive dysfunctions, shown by decreased synaptic protein expression in mice and in glucose-stimulated hippocampal neurons [206]. PACAP serves as a downstream synaptic function-related target gene of the Mas receptor, and the AKT/FOXO1 pathway was identified as a downstream mediator of the Mas receptor in modulating PACAP expression, with FOXO1 binding directly to the PACAP promoter region [206]. This study proves that the already described positive effects of PACAP on cognitive functions could also be present in diabetes-associated cognitive dysfunction [12,207,208]. Bladder dysfunction is also known in diabetes. PACAP plays a role in bladder functions and in the micturition pathway [209,210]. PACAP38 mRNA and PACAP38 peptide expression levels were found to be reduced in type 2 diabetic rats [211]. Sensitivity to PACAP effects was reduced in the testis and uterus of diabetic rats [212,213]. Diabetic gastroenteropathy is another common complication [214,215], and a study in diabetic pigs showed that PACAP-containing enteric neurons reacted sensitively to altered conditions, as immunoreactivity strongly increased in the small intestine, descending colon and stomach [216].

## 9. PACAP and DPPIV

Another very important aspect of PACAP in diabetes and its complications can be the fact that PACAP is cleaved by the dipeptidyl peptidase IV (DPPIV) enzyme, resulting in shorter fragments with decreased antagonist or no biological activity and a half-life of a few minutes [1,66,217,218]. DPPIV inhibitors have now become very important drugs in treatment strategies in diabetes. Two incretin peptides, glucagon-like peptide-1 (GLP-1) and glucose-dependent insulinotropic peptide (GIP), are especially the focus of therapeutic approaches. As these insulinotropic peptides are cleaved by DPPIV, enzyme inhibition is an effective way to elevate the endogenous levels of these peptides. Today, there are numerous compounds available with structural and pharmacokinetic differences. It has now become evident that DPPIV inhibitors not only affect glucose homeostasis, but they also have direct and indirect protective effects in several degenerative and inflammatory conditions [219,220,221]. DPPIV inhibition has been shown to augment insulin response not only to GLP-1 but also to PACAP [222]. Blocking DPPIV can result in higher PACAP concentrations, enabling the peptide to exert long-term cytoprotective effects. This has not been experimentally proven yet, but some data suggest that elevated PACAP levels might add to the beneficial effects of DPPIV inhibitors [188,219,223,224]. Metformin, a glucose-lowering substance mainly acting through decreased hepatic gluconeogenesis and decreased intestinal glucose absorption, is an important medication in type 2 diabetes. The beneficial effects of metformin in degenerative processes have recently been highlighted [225,226,227]. The possible relationship between the PACAP system and metformin was investigated in one study, where it was found that a high-fat diet in mice induced a reduction in the PACAP/VIP system along with neuroinflammation. These alterations could be rescued by metformin administration [228]. Finally, it should be mentioned that not only the cleavage mechanism but also several protective properties of PACAP in diabetic complications are also present in the related peptide, VIP [32,69]. The cytoprotective effects of PACAP, via the specific PAC1 receptor, are stronger in most cases than those of VIP, but the potential therapeutic effects of VIP should also be further explored.

## 10. Conclusions and Future Perspectives

In summary, in the present study, we reviewed data on PACAP being protective in diabetic complications, exerting a multitude of effects in diabetic retinopathy, nephropathy, neuropathy, keratopathy and other accompanying diseases in diabetes. Limitations exist, as in all experimental models, in terms of the translational value of the results. In vitro data use cells deprived from the natural surroundings, and naturally, their behavior can be different from that under in vivo conditions. Animal models represent limitations regarding the species, number of animals, the time-course of diabetes and other factors different from human patients. However, in spite of these limitations, the accumulated data on the protective effects of PACAP in diabetic complications are very promising. The reason why PACAP has not yet been introduced into clinical therapies is multifactorial. Considering that PACAP has very potent protective effects in ischemic and degenerative lesions, it makes one wonder what prevents its clinical use. In an attempt to explore the reasons behind this, we launched a survey among experts in the field and discussed the results in a recent publication [229]. Briefly, the main reasons seem to be its short half-life (5–10 min) and its poor passage through the blood–brain barrier, as well as its wide range of actions. However, we have to keep in mind that the bioavailability and passage properties of PACAP are comparable to those of other neuropeptides. The promising therapeutic effects of PACAP in several pathological conditions have encouraged researchers to design PACAP-related drugs and to develop ways to enhance tissue delivery [2,6,64,229,230,231,232,233,234,235]. The passage and tissue availability through various administration forms have been summarized in detail elsewhere, explaining that PACAP can be effective not only in local or intravenous injections but also in intranasal treatment or eye drops and can represent an easier yet still effective route of administration [234]. Drug development and further experimental efforts will hopefully result in overcoming the hurdles preventing PACAP from being introduced into therapeutic treatments, including diabetes-related conditions [229].

## Figures and Tables

**Table 1:** Main findings in diabetes models on effects of PACAP on ameliorating diabetes/high glucose-induced effects.

Model	PACAP Treatment	PACAP-Induced Effects, Counteracting Diabetes/High Glucose-Induced Effects	Reference
In Vivo Models			
Rat diabetic neuropathy, type 1	PACAP38, 20 µg/100 µL saline i.p., every second day for 8 weeks	- axon–myelin separation ↓ - mitochondrial fission ↓ - unmyelinated fiber atrophy ↓ - endoneurial capillary basement membrane thickening ↓ - mechanical hyperalgesia ↓ - activation of pain processing centers (PAG, dorsal horn) ↓	[33]
Rat diabetic retinopathy, type 1	PACAP38 100 pmol/5 µL saline intravitreal, 3 times during the last week of the 3-week-long survival period	- ganglion cell number ↑ - Müller glial cell overactivation ↓ - cone photoreceptor outer segment degeneration ↓ - dopaminergic amacrine cell damage ↓	[34]
	PACAP38 100 pmol/5 µL saline intravitreal, 3 times during the last week of the 3-week-long survival period	- p-Akt, p-ERK2, PKC and Bcl-2 ↑ - phospho-p38MAPK and caspase-3, -8 and -12 ↓ - number of apoptotic cells ↓	[35]
	PACAP38 100 pmol/5 µL saline intravitreal, 3 times during the last week of the 3-week-long survival period	- photoreceptor, pigment epithelial cell and outer limiting membrane damage ↓ - GLUT1 immunoreactivity ↑ - RAGE mRNA ↓ - number of cone bipolar and ganglion cells ↑ - PAC1, VPAC1, VPAC2 receptor expression ↑ - degeneration of ribbon synapses ↓	[36]
	Intraocular PACAP38, 100 µg/4 µL 1 week after STZ	- HIF-1alpha and HIF-2alpha ↓ - HIF-3alpha ↑	[37]
	Intraocular PACAP38 100 µM, 1 week after STZ	- ADNP expression ↑	[38]
	Intraocular PACAP38 100 µM in 4 µL, 1 week after STZ	- interleukin-1 beta ↓ - VEGF, VEGF receptor expression ↓	[39]
	Intravitreal PACAP 100 pmol/5 µL saline, once a week for 3 weeks in spontaneously hypertensive rats alone or in combination with the PARP inhibitor olaparib	- total retinal thickness ↑ - thickness of ONL, OPL, INL, IPL ↑ - number of ganglion cells ↑ - rod bipolar cell degeneration ↓ - calbindin staining ↑	[40]
	Single intravitreal injection of PACAP38—100 µL, 4 µL	- Bcl-2, p53 ↑	[41]
Rat diabetic retinopathy, type 2	PACAP eye drops (1 µg/drop) twice a day for 16 weeks	- visual function (ERG: a-wave, b-wave, oscillatory potential) ↑ - retinal thickness ↑ - microvascular lesion ↓ - number of surviving pericytes ↑ - number of acellular capillaries ↓ - vessel density ↑	[42]
Rat diabetic nephropathy, type 1	Continuous intravenous treatment with jugular osmotic minipump for 2 weeks	- glomerular damage and vacuolar appearance ↓ - TNF-alpha, TGF-beta ↓ - body weight ↑ - kidney weight, glomerular enlargement ↓ - hyperglycemia, proteinuria, polyuria ↓	[43,44]
	PACAP38, 20 µg/100 µL saline i.p., every second day for 8 weeks	- histological signs of diabetes: intraglomerular PAS positive area expansion, tubular glycogen deposits, arteriolar hyalinosis ↓ - cytokine activation (CINC-1, TIMP-1, LIX, MIG, s-ICAM) ↓	[45]
	PACAP38, 20 µg/100 µL saline i.p., every second day for 8 weeks	- p38 MAPK, cleaved caspase-3 ↓ - NFκB ↓ - pAkt, ERK1/2 ↑ - collagen IV, TGF-beta1 ↓ - glutathione ↑ - glomerular basement membrane thickening ↓	[46]
**High glucose exposure in vitro**			
Retinal explant	100 nM PACAP in vitro on retinal explant exposed to high glucose	- caspase-3 ↓ - VEGF ↓	[47]
ARPE19 pigment epithelial cells exposed to hyperglycemic/hypoxic insult	100 nM PACAP38	- ADNP ↑ - VEGF ↓ - outer retinal barrier permeability ↓ - junctional protein expression (ZO-1, occludin) ↑ - choriocapillaris neovascularization ↓	[38]
ARPE19 pigment epithelial cells exposed to hyperglycemic/hypoxic insult	100 nM PACAP38	- HIF-1alpha ↓ - HIF-3alpha ↑ - VEGF, VEGF receptors ↓ - p38 MAPK ↓	[48,49]
ARPE19 cells exposed to high glucose and interleukin-1 beta	100 nM PACAP38	- hyperpermeability ↓	[50]
Human corneal epithelial cells exposed to high glucose	0.1, 1, 5 µM PACAP38	- cell proliferation, migration ↑ - Ki-67, Bcl-2 mRNA ↑ - autophagy ↑ - p-AMPK, p-ERK, Bcl-2 ↑ - p62 ↓	[51]
Rabbit corneal epithelial cells exposed to high glucose	100 nM PACAP38	- cell viability ↑ - corneal epithelial wound healing ↑ - EGFR, ERK1/2 ↑	[52]
Rabbit corneal epithelial cells exposed to high glucose	100 nM PACAP38	- IL-1beta, TNF-alpha ↓ - activation of NF-kB ↓ - epithelial morphology, corneal barrier thickness ↑	[53]

## Data Availability

No new data were created or analyzed in this study. Data sharing is not applicable to this article.

## References

[1] Vaudry D., Falluel-Morel A., Bourgault S., Basille M., Burel D., Wurtz O., Fournier A., Chow B.K.C., Hashimoto H., Galas L. (2009). Pituitary Adenylate Cyclase-Activating Polypeptide and Its Receptors: 20 Years after the Discovery. Pharmacol. Rev..

[2] Lu J., Piper S.J., Zhao P., Miller L.J., Wootten D., Sexton P.M. (2022). Targeting VIP and PACAP Receptor Signaling: New Insights into Designing Drugs for the PACAP Subfamily of Receptors. Int. J. Mol. Sci..

[3] Rajbhandari A.K., Barson J.R., Gilmartin M.R., Hammack S.E., Chen B.K. (2023). The Functional Heterogeneity of PACAP: Stress, Learning, and Pathology. Neurobiol. Learn. Mem..

[4] Liao C., May V., Li J. (2019). PAC1 Receptors: Shapeshifters in Motion. J. Mol. Neurosci..

[5] Liao C., de Molliens M.P., Schneebeli S.T., Brewer M., Song G., Chatenet D., Braas K.M., May V., Li J. (2019). Targeting the PAC1 Receptor for Neurological and Metabolic Disorders. Curr. Top. Med. Chem..

[6] Tasma Z., Hay D.L. (2025). Decoding PACAP Signaling: Splice Variants, Pathways and Designer Drugs. Cephalalgia.

[7] Langer I., Jeandriens J., Couvineau A., Sanmukh S., Latek D. (2022). Signal Transduction by VIP and PACAP Receptors. Biomedicines.

[8] Hashimoto H., Shintani N., Tanaka K., Mori W., Hirose M., Matsuda T., Sakaue M., Miyazaki J., Niwa H., Tashiro F. (2001). Altered Psychomotor Behaviors in Mice Lacking Pituitary Adenylate Cyclase-Activating Polypeptide (PACAP). Proc. Natl. Acad. Sci. USA.

[9] Seligowski A.V., Clancy K.J., Akman E., Lewis M., May V., Ravichandran C., Jobson S.A., Bradford D.E., Hammack S.E., Carlezon W.A. (2025). Associations between PACAP Levels and Psychophysiological Indicators of Fear and Arousal in Adults with Posttraumatic Stress Symptoms. J. Mood Anxiety Disord..

[10] Singh A., Shim P., Naeem S., Rahman S., Lutfy K. (2025). Pituitary Adenylyl Cyclase-Activating Polypeptide Modulates the Stress Response: The Involvement of Different Brain Areas and Microglia. Front. Psychiatry.

[11] Shintani Y., Hayata-Takano A., Takasaki I., Kurihara T., Miyata A., Yamano Y., Ikuta M., Takeshita R., Murata K., Oguri T. (2025). Rapid and Long-Lasting Antidepressant-like Effects of the Pituitary Adenylate Cyclase-Activating Polypeptide Receptor Antagonist PA-915 in Chronic Stress Mouse Models. Mol. Psychiatry.

[12] Ciranna L., Costa L. (2019). Pituitary Adenylate Cyclase-Activating Polypeptide Modulates Hippocampal Synaptic Transmission and Plasticity: New Therapeutic Suggestions for Fragile X Syndrome. Front. Cell. Neurosci..

[13] Garami A., Pakai E., Rumbus Z., Solymar M., Reglodi D., Tamas A. (2016). The Role of PACAP in the Regulation of Body Temperature. Pituitary Adenylate Cyclase Activating Polypeptide—PACAP.

[14] Tóth D., Simon G., Reglődi D. (2023). Pituitary Adenylate Cyclase-Activating Polypeptide (PACAP) and Sudden Infant Death Syndrome: A Potential Model for Investigation. Int. J. Mol. Sci..

[15] Incognito A.V., Barioni N.O., Sharkey K.A., Wilson R.J.A. (2025). Carotid Body Stress and Inflammatory Mediators Uniformly Excite Autonomic Output. Circ. Res..

[16] Barrett K.T., Hasan S.U., Scantlebury M.H., Wilson R.J.A. (2021). Impaired Cardiorespiratory Responses to Hypercapnia in Neonatal Mice Lacking PAC1 but Not VPAC2 Receptors. Am. J. Physiol. Regul. Integr. Comp. Physiol..

[17] Rácz K., Segal Y., Lénárt K., Fillér C., Tóth A., Szegeczki V., Gergely P., Zákány R., Reglődi D., Juhász T. (2025). Cartilage Degradation Is Followed by PAC1 Receptor Reduction in Articular Cartilage of Human Knee Joints. Geroscience.

[18] Sun Z.-P., Wu S.-P., Liang C.-D., Zhao C.-X., Sun B.-Y. (2019). The Synovial Fluid Neuropeptide PACAP May Act as a Protective Factor during Disease Progression of Primary Knee Osteoarthritis and Is Increased Following Hyaluronic Acid Injection. Innate Immun..

[19] Diener H.C. (2025). PACAP and Migraine. Brain.

[20] Reglodi D., Tamas A., Jungling A., Vaczy A., Rivnyak A., Fulop B.D., Szabo E., Lubics A., Atlasz T. (2018). Protective Effects of Pituitary Adenylate Cyclase Activating Polypeptide against Neurotoxic Agents. Neurotoxicology.

[21] Reglodi D., Kiss P., Lubics A., Tamas A. (2011). Review on the Protective Effects of PACAP in Models of Neurodegenerative Diseases In Vitro and In Vivo. Curr. Pharm. Des..

[22] Reglodi D., Kiss P., Szabadfi K., Atlasz T., Gabriel R., Horvath G., Szakaly P., Sandor B., Lubics A., Laszlo E. (2012). PACAP Is an Endogenous Protective Factor—Insights from PACAP-Deficient Mice. J. Mol. Neurosci..

[23] Falluel-Morel A., Aubert N., Vaudry D., Desfeux A., Allais A., Burel D., Basille M., Vaudry H., Laudenbach V., Gonzalez B.J. (2008). Interactions of PACAP and Ceramides in the Control of Granule Cell Apoptosis During Cerebellar Development. J. Mol. Neurosci..

[24] Abad C., Tan Y.-V. (2018). Immunomodulatory Roles of PACAP and VIP: Lessons from Knockout Mice. J. Mol. Neurosci..

[25] Li H., Cao L., Yi P.-Q., Xu C., Su J., Chen P.-Z., Li M., Chen J.-Y. (2019). Pituitary Adenylate Cyclase-Activating Polypeptide Ameliorates Radiation-Induced Cardiac Injury. Am. J. Transl. Res..

[26] Gomariz R.P., Juarranz Y., Abad C., Arranz A., Leceta J., Martinez C. (2006). VIP–PACAP System in Immunity. Ann. New York Acad. Sci..

[27] Tang Y., Lv B., Wang H., Xiao X., Zuo X. (2008). PACAP Inhibit the Release and Cytokine Activity of HMGB1 and Improve the Survival during Lethal Endotoxemia. Int. Immunopharmacol..

[28] Mori H., Nakamachi T., Ohtaki H., Yofu S., Sato A., Endo K., Iso Y., Suzuki H., Takeyama Y., Shintani N. (2010). Cardioprotective Effect of Endogenous Pituitary Adenylate Cyclase-Activating Polypeptide on Doxorubicin-Induced Cardiomyopathy in Mice. Circ. J..

[29] Padua D., Vu J.P., Germano P.M., Pisegna J.R. (2016). The Role of Neuropeptides in Mouse Models of Colitis. J. Mol. Neurosci..

[30] Ohtaki H., Nakamachi T., Dohi K., Shioda S. (2008). Role of PACAP in Ischemic Neural Death. J. Mol. Neurosci..

[31] Marzagalli R., Scuderi S., Drago F., Waschek J.A., Castorina A. (2015). Emerging Role of PACAP as a New Potential Therapeutic Target in Major Diabetes Complications. Int. J. Endocrinol..

[32] Wen S., Yuan Y., Li Y., Xu C., Chen L., Ren Y., Wang C., He Y., Li X., Gong M. (2025). The Effects of Non-Insulin Anti-Diabetic Medications on the Diabetic Microvascular Complications: A Systematic Review and Meta-Analysis of Randomized Clinical Trials. BMC Endocr. Disord..

[33] Kiss P., Banki E., Gaszner B., Nagy D., Helyes Z., Pal E., Reman G., Toth G., Tamas A., Reglodi D. (2021). Protective Effects of PACAP in a Rat Model of Diabetic Neuropathy. Int. J. Mol. Sci..

[34] Szabadfi K., Atlasz T., Kiss P., Reglodi D., Szabo A., Kovacs K., Szalontai B., Setalo G., Banki E., Csanaky K. (2012). Protective Effects of the Neuropeptide PACAP in Diabetic Retinopathy. Cell Tissue Res..

[35] Szabadfi K., Szabo A., Kiss P., Reglodi D., Setalo G., Kovacs K., Tamas A., Toth G., Gabriel R. (2014). PACAP Promotes Neuron Survival in Early Experimental Diabetic Retinopathy. Neurochem. Int..

[36] Szabadfi K., Reglodi D., Szabo A., Szalontai B., Valasek A., Setalo G., Kiss P., Tamas A., Wilhelm M., Gabriel R. (2016). Pituitary Adenylate Cyclase Activating Polypeptide, A Potential Therapeutic Agent for Diabetic Retinopathy in Rats: Focus on the Vertical Information Processing Pathway. Neurotox. Res..

[37] D’Amico A.G., Maugeri G., Reitano R., Bucolo C., Saccone S., Drago F., D’Agata V. (2015). PACAP Modulates Expression of Hypoxia-Inducible Factors in Streptozotocin-Induced Diabetic Rat Retina. J. Mol. Neurosci..

[38] D’Amico A.G., Maugeri G., Magrì B., Lombardo C., Saccone S., Federico C., Cavallaro P., Giunta S., Bucolo C., D’Agata V. (2023). PACAP-ADNP Axis Prevents Outer Retinal Barrier Breakdown and Choroidal Neovascularization by Interfering with VEGF Secreted from Retinal Pigmented Epitelium Cells. Peptides.

[39] D’Amico A.G., Maugeri G., Rasà D.M., Bucolo C., Saccone S., Federico C., Cavallaro S., D’Agata V. (2017). Modulation of IL-1β and VEGF Expression in Rat Diabetic Retinopathy after PACAP Administration. Peptides.

[40] Pöstyéni E., Szabadfi K., Sétáló G., Gabriel R. (2021). A Promising Combination: PACAP and PARP Inhibitor Have Therapeutic Potential in Models of Diabetic and Hypertensive Retinopathies. Cells.

[41] Giunta S., Castorina A., Bucolo C., Magro G., Drago F., D’Agata V. (2012). Early Changes in Pituitary Adenylate Cyclase-Activating Peptide, Vasoactive Intestinal Peptide and Related Receptors Expression in Retina of Streptozotocin-Induced Diabetic Rats. Peptides.

[42] Li L., Patko E., Szabo E., Molitor D., Meresz B., Reglodi D., Varga A., Denes D., Dai L., Wang H. (2025). The Protective Effect of Topical PACAP38 in Retinal Morphology and Function of Type 2 Diabetic Retinopathy. Int. J. Mol. Sci..

[43] Arimura A., Li M., Batuman V. (2006). Treatment of Renal Failure Associated with Multiple Myeloma and Other Diseases by PACAP-38. Ann. N. Y. Acad. Sci..

[44] Li M., Maderdrut J.L., Lertora J.J.L., Arimura A., Batuman V. (2008). Renoprotection by Pituitary Adenylate Cyclase-Activating Polypeptide in Multiple Myeloma and Other Kidney Diseases. Regul. Pept..

[45] Banki E., Degrell P., Kiss P., Kovacs K., Kemeny A., Csanaky K., Duh A., Nagy D., Toth G., Tamas A. (2013). Effect of PACAP Treatment on Kidney Morphology and Cytokine Expression in Rat Diabetic Nephropathy. Peptides.

[46] Banki E., Kovacs K., Nagy D., Juhasz T., Degrell P., Csanaky K., Kiss P., Jancso G., Toth G., Tamas A. (2014). Molecular Mechanisms Underlying the Nephroprotective Effects of PACAP in Diabetes. J. Mol. Neurosci..

[47] Amato R., Biagioni M., Cammalleri M., Dal Monte M., Casini G. (2016). VEGF as a Survival Factor in Ex Vivo Models of Early Diabetic Retinopathy. Investig. Ophthalmol. Vis. Sci..

[48] Maugeri G., D’Amico A.G., Gagliano C., Saccone S., Federico C., Cavallaro S., D’Agata V. (2017). VIP Family Members Prevent Outer Blood Retinal Barrier Damage in a Model of Diabetic Macular Edema. J. Cell. Physiol..

[49] Maugeri G., D’Amico A.G., Saccone S., Federico C., Cavallaro S., D’Agata V. (2017). PACAP and VIP Inhibit HIF-1α-mediated VEGF Expression in a Model of Diabetic Macular Edema. J. Cell. Physiol..

[50] Scuderi S., D’Amico A.G., Castorina A., Imbesi R., Carnazza M.L., D’Agata V. (2013). Ameliorative Effect of PACAP and VIP against Increased Permeability in a Model of Outer Blood Retinal Barrier Dysfunction. Peptides.

[51] Bao Y., Li B. (2025). Protective Effects of Pituitary Adenylate Cyclase-Activating Peptide (PACAP) on High Glucose-Induced Damage in Human Corneal Epithelial Cell. BMC Ophthalmol..

[52] Maugeri G., D’Amico A.G., Magrì B., Giunta S., Saccone S., Federico C., Bucolo C., Musumeci G., D’Agata V. (2023). Protective Effect of Pituitary Adenylate Cyclase Activating Polypeptide in Diabetic Keratopathy. Peptides.

[53] Maugeri G., D’Amico A.G., Palmeri N., Pricoco E., Brancato D., Federico C., D’Agata V. (2025). Protective Effects of PACAP against High Glucose-Induced Inflammation on Air-Liquid Interface Corneal Epithelium Barrier. Peptides.

[54] Ferencz S., Reglodi D., Kaszas B., Bardosi A., Toth D., Vekony Z., Vicena V., Karadi O., Kelemen D. (2019). PACAP and PAC1 Receptor Expression in Pancreatic Ductal Carcinoma. Oncol. Lett..

[55] Muroi M., Shioda S., Yada T., Zhou C.J., Nakai Y., Nakajo S., Arimura A. (1998). Distribution and Ultrastructural Localization of PACAP Receptors in the Rat Pancreatic Islets. Ann. N. Y. Acad. Sci..

[56] Li W., Yu G., Liu Y., Sha L. (2019). Intrapancreatic Ganglia and Neural Regulation of Pancreatic Endocrine Secretion. Front. Neurosci..

[57] Winzell M.S., Ahrén B. (2007). G-Protein-Coupled Receptors and Islet Function—Implications for Treatment of Type 2 Diabetes. Pharmacol. Ther..

[58] Ferencz S., Toth D., Kaszas B., Bardosi S., Vicena V., Karadi O., Reglodi D., Kelemen D. (2021). PACAP and PAC1 Receptor Expression in Human Insulinomas. Int. J. Pept. Res. Ther..

[59] Cavestro C. (2025). Metabolic Dysfunction and Dietary Interventions in Migraine Management: The Role of Insulin Resistance and Neuroinflammation—A Narrative and Scoping Review. Brain Sci..

[60] Filipsson K., Kvist-Reimer M., Ahrén B. (2001). The Neuropeptide Pituitary Adenylate Cyclase–Activating Polypeptide and Islet Function. Diabetes.

[61] Filipsson K., Tornøe K., Holst J., Ahrén B. (1997). Pituitary Adenylate Cyclase-Activating Polypeptide Stimulates Insulin and Glucagon Secretion in Humans. J. Clin. Endocrinol. Metab..

[62] Winzell M.S., Ahrén B. (2007). Role of VIP and PACAP in Islet Function. Peptides.

[63] Nakata M., Yada T. (2004). Physiological and Therapeutic Roles of PACAP in Glucose Metabolism and Diabetes. Folia Pharmacol. Jpn..

[64] Sanlioglu A.D., Karacay B., Balci M.K., Griffith T.S., Sanlioglu S. (2012). Therapeutic Potential of VIP vs PACAP in Diabetes. J. Mol. Endocrinol..

[65] Moody T.W., Ito T., Osefo N., Jensen R.T. (2011). VIP and PACAP: Recent Insights into Their Functions/Roles in Physiology and Disease from Molecular and Genetic Studies. Curr. Opin. Endocrinol. Diabetes Obes..

[66] Green B.D., Irwin N., Flatt P.R. (2006). Pituitary Adenylate Cyclase-Activating Peptide (PACAP): Assessment of Dipeptidyl Peptidase IV Degradation, Insulin-Releasing Activity and Antidiabetic Potential. Peptides.

[67] Persson-Sjögren S., Forsgren S., Lindström P. (2006). Vasoactive Intestinal Polypeptide and Pituitary Adenylate Cyclase Activating Polypeptide: Effects on Insulin Release in Isolated Mouse Islets in Relation to Metabolic Status and Age. Neuropeptides.

[68] Yada T., Sakurada M., Ihida K., Nakata M., Murata F., Arimura A., Kikuchi M. (1994). Pituitary Adenylate Cyclase Activating Polypeptide Is an Extraordinarily Potent Intra-Pancreatic Regulator of Insulin Secretion from Islet Beta-Cells. J. Biol. Chem..

[69] Bertrand G., Puech R., Maisonnasse Y., Bockaert J., Loubatières-Mariani M.M. (1996). Comparative Effects of PACAP and VIP on Pancreatic Endocrine Secretions and Vascular Resistance in Rat. Br. J. Pharmacol..

[70] Green B.D., Irwin N., Cassidy R.S., Gault V.A., Flatt P.R. (2006). Long-Term Administration of PACAP Receptor Antagonist, PACAP(6-27), Impairs Glucose Tolerance and Insulin Sensitivity in Obese Diabetic Ob/Ob Mice. Peptides.

[71] Borboni P., Porzio O., Pierucci D., Cicconi S., Magnaterra R., Federici M., Sesti G., Lauro D., D’Agata V., Cavallaro S. (1999). Molecular and Functional Characterization of Pituitary Adenylate Cyclase-Activating Polypeptide (PACAP-38)/Vasoactive Intestinal Polypeptide Receptors in Pancreatic β-Cells and Effects of PACAP-38 on Components of the Insulin Secretory System. Endocrinology.

[72] Yokota C., Kawai K., Ohashi S., Watanabe Y., Yamashita K. (1995). PACAP Stimulates Glucose Output from the Perfused Rat Liver. Peptides.

[73] Sekiguchi Y., Kasai K., Hasegawa K., Suzuki Y., Shimoda S.-I. (1994). Glycogenolytic Activity of Pituitary Adenylate Cyclase Activating Polypeptide (PACAP) in Vivo and in Vitro. Life Sci..

[74] Carbone E. (2024). TRPC5: A New Entry to the Chromaffin Cell’s Palette of Ion Channels That Control Adrenal Response to Hypoglycemia. EMBO J..

[75] Khodai T., Nunn N., Worth A.A., Feetham C.H., Belle M.D.C., Piggins H.D., Luckman S.M. (2018). PACAP Neurons in the Ventromedial Hypothalamic Nucleus Are Glucose Inhibited and Their Selective Activation Induces Hyperglycaemia. Front. Endocrinol..

[76] Meng A., Ameroso D., Rios M. (2023). MGluR5 in Astrocytes in the Ventromedial Hypothalamus Regulates Pituitary Adenylate Cyclase-Activating Polypeptide Neurons and Glucose Homeostasis. J. Neurosci..

[77] Yi C.-X., Sun N., Ackermans M.T., Alkemade A., Foppen E., Shi J., Serlie M.J., Buijs R.M., Fliers E., Kalsbeek A. (2010). Pituitary Adenylate Cyclase-Activating Polypeptide Stimulates Glucose Production via the Hepatic Sympathetic Innervation in Rats. Diabetes.

[78] Portela-Gomes G.M., Lukinius A., Ljungberg O., Efendic S., Ahrén B., Abdel-Halim S.M. (2003). PACAP Is Expressed in Secretory Granules of Insulin and Glucagon Cells in Human and Rodent Pancreas. Regul. Pept..

[79] Yu R., Wang J., Li J., Wang Y., Zhang H., Chen J., Huang L., Liu X. (2010). A Novel Cyclopeptide from the Cyclization of PACAP(1–5) with Potent Activity towards PAC1 Attenuates STZ-Induced Diabetes. Peptides.

[80] Ma Y., Fang S., Zhao S., Wang X., Wang D., Ma M., Luo T., Hong A. (2015). A Recombinant Slow-Release PACAP-Derived Peptide Alleviates Diabetes by Promoting Both Insulin Secretion and Actions. Biomaterials.

[81] Sakuma Y., Ricordi C., Miki A., Yamamoto T., Mita A., Barker S., Damaris R.M., Pileggi A., Yasuda Y., Yada T. (2009). Effect of Pituitary Adenylate Cyclase–Activating Polypeptide in Islet Transplantation. Transplant. Proc..

[82] Prevost G., Arabo A., Jian L., Quelennec E., Cartier D., Hassan S., Falluel-Morel A., Tanguy Y., Gargani S., Lihrmann I. (2013). The PACAP-Regulated Gene Selenoprotein T Is Abundantly Expressed in Mouse and Human β-Cells and Its Targeted Inactivation Impairs Glucose Tolerance. Endocrinology.

[83] Firdos F., Pramanik T., Mittal A. (2025). Temporal Effects of a Viral Peptide on Glucose-Stimulated Insulin Secretion. ACS Infect. Dis..

[84] Sakurai Y., Inoue H., Shintani N., Arimori A., Hamagami K., Hayata-Takano A., Baba A., Hashimoto H. (2012). Compensatory Recovery of Blood Glucose Levels in KKAy Mice Fed a High-Fat Diet: Insulin-Sparing Effects of PACAP Overexpression in β Cells. J. Mol. Neurosci..

[85] Tomimoto S., Hashimoto H., Shintani N., Yamamoto K., Kawabata Y., Hamagami K.-I., Yamagata K., Miyagawa J.-I., Baba A. (2004). Overexpression of Pituitary Adenylate Cyclase-Activating Polypeptide in Islets Inhibits Hyperinsulinemia and Islet Hyperplasia in Agouti Yellow Mice. J. Pharmacol. Exp. Ther..

[86] Yamamoto K., Hashimoto H., Tomimoto S., Shintani N., Miyazaki J., Tashiro F., Aihara H., Nammo T., Li M., Yamagata K. (2003). Overexpression of PACAP in Transgenic Mouse Pancreatic β-Cells Enhances Insulin Secretion and Ameliorates Streptozotocin-Induced Diabetes. Diabetes.

[87] Tsunekawa S., Miura Y., Yamamoto N., Itoh Y., Ariyoshi Y., Senda T., Oiso Y., Niki I. (2005). Systemic Administration of Pituitary Adenylate Cyclase-Activating Polypeptide Maintains Beta-Cell Mass and Retards Onset of Hyperglycaemia in Beta-Cell-Specific Calmodulin-Overexpressing Transgenic Mice. Eur. J. Endocrinol..

[88] Yada T., Sakurada M., Filippson K., Kikuchi M., Ahrén B. (2000). Intraperitoneal PACAP Administration Decreases Blood Glucose in GK Rats, and in Normal and High Fat Diet Mice. Ann. N. Y. Acad. Sci..

[89] Gray S.L., Cummings K.J., Jirik F.R., Sherwood N.M. (2001). Targeted Disruption of the Pituitary Adenylate Cyclase-Activating Polypeptide Gene Results in Early Postnatal Death Associated with Dysfunction of Lipid and Carbohydrate Metabolism. Mol. Endocrinol..

[90] Adams B.A., Gray S.L., Isaac E.R., Bianco A.C., Vidal-Puig A.J., Sherwood N.M. (2008). Feeding and Metabolism in Mice Lacking Pituitary Adenylate Cyclase-Activating Polypeptide. Endocrinology.

[91] Bozadjieva-Kramer N., Ross R.A., Johnson D.Q., Fenselau H., Haggerty D.L., Atwood B., Lowell B., Flak J.N. (2021). The Role of Mediobasal Hypothalamic PACAP in the Control of Body Weight and Metabolism. Endocrinology.

[92] Tomimoto S., Ojika T., Shintani N., Hashimoto H., Hamagami K., Ikeda K., Nakata M., Yada T., Sakurai Y., Shimada T. (2008). Markedly Reduced White Adipose Tissue and Increased Insulin Sensitivity in Adcyap1-Deficient Mice. J. Pharmacol. Sci..

[93] Reglodi D., Jungling A., Longuespée R., Kriegsmann J., Casadonte R., Kriegsmann M., Juhasz T., Bardosi S., Tamas A., Fulop B.D. (2018). Accelerated Pre-Senile Systemic Amyloidosis in PACAP Knockout Mice—A Protective Role of PACAP in Age-Related Degenerative Processes. J. Pathol..

[94] Jamen F., Persson K., Bertrand G., Rodriguez-Henche N., Puech R., Bockaert J., Ahrén B., Brabet P. (2000). PAC1 Receptor–Deficient Mice Display Impaired Insulinotropic Response to Glucose and Reduced Glucose Tolerance. J. Clin. Invest..

[95] Splitthoff P., Rasbach E., Neudert P., Bonaterra G.A., Schwarz A., Mey L., Schwarzbach H., Eiden L.E., Weihe E., Kinscherf R. (2020). PAC1 Deficiency Attenuates Progression of Atherosclerosis in ApoE Deficient Mice under Cholesterol-Enriched Diet. Immunobiology.

[96] Hawke Z., Ivanov T.R., Bechtold D.A., Dhillon H., Lowell B.B., Luckman S.M. (2009). PACAP Neurons in the Hypothalamic Ventromedial Nucleus Are Targets of Central Leptin Signaling. J. Neurosci..

[97] Sekar R., Wang L., Chow B.K.C. (2017). Central Control of Feeding Behavior by the Secretin, PACAP, and Glucagon Family of Peptides. Front. Endocrinol..

[98] Feng J., Chen W., Li S., Fang Q., Chen X., Bai G., Tian M., Huang Y., Xu P., Wang Z. (2024). PACAP Ameliorates Obesity-induced Insulin Resistance through FAIM /Rictor/ AKT Axis. FEBS J..

[99] Basu R., Elmendorf A.J., Lorentz B., Mahler C.A., Lazzaro O., App B., Zhou S., Yamamoto Y., Suber M., Wann J.C. (2024). Ventromedial Hypothalamic Nucleus Subset Stimulates Tissue Thermogenesis via Preoptic Area Outputs. Mol. Metab..

[100] Luo W., Dai J., Liu J., Huang Y., Zheng Z., Xu P., Ma Y. (2022). PACAP Attenuates Hepatic Lipid Accumulation through the FAIM/AMPK/IRβ Axis during Overnutrition. Mol. Metab..

[101] Duesman S.J., Shetty S., Patel S., Ogale N., Mohamed F., Sparman N., Rajbhandari P., Rajbhandari A.K. (2022). Sexually Dimorphic Role of the Locus Coeruleus PAC1 Receptors in Regulating Acute Stress-Associated Energy Metabolism. Front. Behav. Neurosci..

[102] Bakalar D., Gavrilova O., Jiang S.Z., Zhang H., Roy S., Williams S.K., Liu N., Wisser S., Usdin T.B., Eiden L.E. (2023). Constitutive and Conditional Deletion Reveals Distinct Phenotypes Driven by Developmental versus Neurotransmitter Actions of the Neuropeptide PACAP. J. Neuroendocrinol..

[103] Suzuki K., Yamaga H., Ohtaki H., Hirako S., Miyamoto K., Nakamura M., Yanagisawa K., Shimada T., Hosono T., Hashimoto H. (2023). Effect of PACAP on Heat Exposure. Int. J. Mol. Sci..

[104] Yang F., Zhao S., Wang P., Xiang W. (2023). Hypothalamic Neuroendocrine Integration of Reproduction and Metabolism in Mammals. J. Endocrinol..

[105] Hurley M.M., Anderson E.M., Chen C., Maunze B., Hess E.M., Block M.E., Patel N., Cooper Z., McCoy R., Dabra T. (2020). Acute Blockade of PACAP-Dependent Activity in the Ventromedial Nucleus of the Hypothalamus Disrupts Leptin-Induced Behavioral and Molecular Changes in Rats. Neuroendocrinology.

[106] Sayers S., Le N., Wagner E.J. (2024). The Role of Pituitary Adenylate Cyclase-Activating Polypeptide Neurons in the Hypothalamic Ventromedial Nucleus and the Cognate PAC1 Receptor in the Regulation of Hedonic Feeding. Front. Nutr..

[107] Vu J.P., Luong L., Sanford D., Oh S., Kuc A., Pisegna R., Lewis M., Pisegna J.R., Germano P.M. (2023). PACAP and VIP Neuropeptides’ and Receptors’ Effects on Appetite, Satiety and Metabolism. Biology.

[108] Martins A.B., Brownlow M.L., Araújo B.B., Garnica-Siqueira M.C., Zaia D.A.M., Leite C.M., Zaia C.T.B.V., Uchoa E.T. (2022). Arcuate Nucleus of the Hypothalamus Contributes to the Hypophagic Effect and Plasma Metabolic Changes Induced by Vasoactive Intestinal Peptide and Pituitary Adenylate Cyclase-Activating Polypeptide. Neurochem. Int..

[109] Gu H.F. (2002). Genetic Variation Screening and Association Studies of the Adenylate Cyclase Activating Polypeptide 1 (ADCYAP1) Gene in Patients with Type 2 Diabetes. Hum. Mutat..

[110] Yamamoto J., Imai J., Izumi T., Takahashi H., Kawana Y., Takahashi K., Kodama S., Kaneko K., Gao J., Uno K. (2017). Neuronal Signals Regulate Obesity Induced β-Cell Proliferation by FoxM1 Dependent Mechanism. Nat. Commun..

[111] Nakata M., Shintani N., Hashimoto H., Baba A., Yada T. (2010). Intra-Islet PACAP Protects Pancreatic β-Cells Against Glucotoxicity and Lipotoxicity. J. Mol. Neurosci..

[112] Onoue S., Hanato J., Kuriyama K., Mizumoto T., Yamada S. (2011). Development of PACAP38 Analogue with Improved Stability: Physicochemical and In Vitro/In Vivo Pharmacological Characterization. J. Mol. Neurosci..

[113] Oliva-Cárdenas A., Ávalos-Rodríguez A., Díaz-Rosas G., Cruz M., Nario-Chaidez H.F., Contreras-Ramos A., Ortega-Camarillo C. (2025). Conditioned Medium from Adipose Mesenchymal Stromal Cells Stimulated with Pituitary Adenylate Cyclase-Activating Polypeptide (PACAP) Mitigates RINm5F Pancreatic β-Cell Dysfunction. Mol. Biol. Rep..

[114] Hou X., Yang D., Yang G., Li M., Zhang J., Zhang J., Zhang Y., Liu Y. (2022). Therapeutic Potential of Vasoactive Intestinal Peptide and Its Receptor VPAC2 in Type 2 Diabetes. Front. Endocrinol..

[115] Patko E., Szabo E., Toth D., Tornoczky T., Bosnyak I., Vaczy A., Atlasz T., Reglodi D. (2022). Distribution of PACAP and PAC1 Receptor in the Human Eye. J. Mol. Neurosci..

[116] Shioda S., Takenoya F., Wada N., Hirabayashi T., Seki T., Nakamachi T. (2016). Pleiotropic and Retinoprotective Functions of PACAP. Anat. Sci. Int..

[117] Seki T., Shioda S., Ogino D., Nakai Y., Arimura A., Koide R. (1997). Distribution and Ultrastructural Localization of a Receptor for Pituitary Adenylate Cyclase Activating Polypeptide and Its MRNA in the Rat Retina. Neurosci. Lett..

[118] Seki T., Shioda S., Izumi S., Arimura A., Koide R. (2000). Electron Microscopic Observation of Pituitary Adenylate Cyclase-Activating Polypeptide (PACAP)-Containing Neurons in the Rat Retina. Peptides.

[119] Seki T., Shioda S., Nakai Y., Arimura A., Koide R. (1998). Distribution and Ultrastractural Localization of Pituitary Adenylate Cyclase-Activating Polypeptide (PACAP) and Its Receptor in the Rat Retina. Ann. N. Y. Acad. Sci..

[120] Izumi S., Seki T., Shioda S., Zhou C.-J., Arimura A., Koide R. (2000). Ultrastructural Localization of PACAP Immunoreactivity in the Rat Retina. Ann. N. Y. Acad. Sci..

[121] Seki T., Izumi S., Shioda S., Zhou C.J., Arimura A., Koide R. (2000). Gene Expression for PACAP Receptor MRNA in the Rat Retina by in Situ Hybridization and in Situ RT-PCR. Ann. N. Y. Acad. Sci..

[122] Atlasz T., Vaczy A., Werling D., Kiss P., Tamas A., Kovacs K., Fabian E., Kvarik T., Mammel B., Danyadi B., Reglodi D., Tamas A. (2016). Protective Effects of PACAP in the Retina. Pituitary Adenylate Cyclase Activating Polypeptide—PACAP.

[123] Pöstyéni E., Kovács-Valasek A., Dénes V., Mester A., Sétáló G., Gábriel R. (2021). PACAP for Retinal Health: Model for Cellular Aging and Rescue. Int. J. Mol. Sci..

[124] Kovács-Valasek A., Szabadfi K., Dénes V., Szalontai B., Tamás A., Kiss P., Szabó A., Setalo Jr G., Reglődi D., Gábriel R. (2017). Accelerated Retinal Aging in PACAP Knock-out Mice. Neuroscience.

[125] Szabadfi K., Atlasz T., Kiss P., Danyadi B., Tamas A., Helyes Z., Hashimoto H., Shintani N., Baba A., Toth G. (2012). Mice Deficient in Pituitary Adenylate Cyclase Activating Polypeptide (PACAP) Are More Susceptible to Retinal Ischemic Injury In Vivo. Neurotox. Res..

[126] Kvarik T., Reglodi D., Werling D., Vaczy A., Kovari P., Szabo E., Kovacs K., Hashimoto H., Ertl T., Gyarmati J. (2021). The Protective Effects of Endogenous PACAP in Oxygen-Induced Retinopathy. J. Mol. Neurosci..

[127] D’Amico A.G., Maugeri G., Musumeci G., Reglodi D., D’Agata V. (2021). PACAP and NAP: Effect of Two Functionally Related Peptides in Diabetic Retinopathy. J. Mol. Neurosci..

[128] Nakatani M., Seki T., Shinohara Y., Taki C., Nishimura S., Takaki A., Shioda S. (2006). Pituitary Adenylate Cyclase-Activating Peptide (PACAP) Stimulates Production of Interleukin-6 in Rat Müller Cells. Peptides.

[129] Szabadfi K., Pinter E., Reglodi D., Gabriel R. (2014). Neuropeptides, Trophic Factors, and Other Substances Providing Morphofunctional and Metabolic Protection in Experimental Models of Diabetic Retinopathy. Int. Rev. Cell Mol. Biol..

[130] Gábriel R. (2013). Neuropeptides and Diabetic Retinopathy. Br. J. Clin. Pharmacol..

[131] Horvath G., Reglodi D., Fabian E., Opper B. (2022). Effects of Pituitary Adenylate Cyclase Activating Polypeptide on Cell Death. Int. J. Mol. Sci..

[132] Allais A., Burel D., Roy V., Arthaud S., Galas L., Isaac E.R., Desfeux A., Parent B., Fournier A., Chapillon P. (2010). Balanced Effect of PACAP and FasL on Granule Cell Death during Cerebellar Development: A Morphological, Functional and Behavioural Characterization. J. Neurochem..

[133] Ji H., Zhang Y., Shen X., Gao F., Huang C.Y., Abad C., Busuttil R.W., Waschek J.A., Kupiec-Weglinski J.W. (2013). Neuropeptide PACAP in Mouse Liver Ischemia and Reperfusion Injury: Immunomodulation by the CAMP-PKA Pathway. Hepatology.

[134] Hartfield P.J., Bilney A.J., Murray A.W. (1998). Neurotrophic Factors Prevent Ceramide-Induced Apoptosis Downstream of C-Jun N-Terminal Kinase Activation in PC12 Cells. J. Neurochem..

[135] Roth E., Weber G., Kiss P., Horváth G., Toth G., Gasz B., Ferencz A., Gallyas F., Reglodi D., Racz B. (2009). Effects of PACAP and Preconditioning against Ischemia/Reperfusion-induced Cardiomyocyte Apoptosis In Vitro. Ann. N. Y. Acad. Sci..

[136] Masmoudi-Kouki O., Douiri S., Hamdi Y., Kaddour H., Bahdoudi S., Vaudry D., Basille M., Leprince J., Fournier A., Vaudry H. (2011). Pituitary Adenylate Cyclase-Activating Polypeptide Protects Astroglial Cells against Oxidative Stress-Induced Apoptosis. J. Neurochem..

[137] Morio H., Tatsuno I., Tanaka T., Uchida D., Hirai A., Tamura Y., Saito Y. (2006). Pituitary Adenylate Cyclase-Activating Polypeptide (PACAP) Is a Neurotrophic Factor for Cultured Rat Cortical Neurons. Ann. N. Y. Acad. Sci..

[138] Vaudry D., Rousselle C., Basille M., Falluel-Morel A., Pamantung T.F., Fontaine M., Fournier A., Vaudry H., Gonzalez B.J. (2002). Pituitary Adenylate Cyclase-Activating Polypeptide Protects Rat Cerebellar Granule Neurons against Ethanol-Induced Apoptotic Cell Death. Proc. Natl. Acad. Sci. USA.

[139] Mei Y.A., Vaudry D., Basille M., Castel H., Fournier A., Vaudry H., Gonzalez B.J. (2004). PACAP Inhibits Delayed Rectifier Potassium Current via a CAMP/PKA Transduction Pathway: Evidence for the Involvement of I k in the Anti-apoptotic Action of PACAP. Eur. J. Neurosci..

[140] Tanaka J., Koshimura K., Murakami Y., Sohmiya M., Yanaihara N., Kato Y. (1997). Neuronal Protection from Apoptosis by Pituitary Adenylate Cyclase-Activating Polypeptide. Regul. Pept..

[141] Douiri S., Bahdoudi S., Hamdi Y., Cubì R., Basille M., Fournier A., Vaudry H., Tonon M., Amri M., Vaudry D. (2016). Involvement of Endogenous Antioxidant Systems in the Protective Activity of Pituitary Adenylate Cyclase-activating Polypeptide against Hydrogen Peroxide-induced Oxidative Damages in Cultured Rat Astrocytes. J. Neurochem..

[142] Nakamachi T., Li M., Shioda S., Arimura A. (2006). Signaling Involved in Pituitary Adenylate Cyclase-Activating Polypeptide-Stimulated ADNP Expression. Peptides.

[143] Szabo E., Patko E., Vaczy A., Molitor D., Csutak A., Toth G., Reglodi D., Atlasz T. (2021). Retinoprotective Effects of PACAP Eye Drops in Microbead-Induced Glaucoma Model in Rats. Int. J. Mol. Sci..

[144] Werling D., Banks W., Salameh T., Kvarik T., Kovacs L., Vaczy A., Szabo E., Mayer F., Varga R., Tamas A. (2017). Passage through the Ocular Barriers and Beneficial Effects in Retinal Ischemia of Topical Application of PACAP1-38 in Rodents. Int. J. Mol. Sci..

[145] Fabian E., Reglodi D., Horvath G., Opper B., Toth G., Fazakas C., Vegh A.G., Wilhelm I., Krizbai I.A. (2019). Pituitary Adenylate Cyclase Activating Polypeptide Acts against Neovascularization in Retinal Pigment Epithelial Cells. Ann. N. Y. Acad. Sci..

[146] Fábián E., Horváth G., Opper B., Atlasz T., Tóth G., Reglődi D. (2021). PACAP Is Protective Against Cellular Stress in Retinal Pigment Epithelial Cells. Int. J. Pept. Res. Ther..

[147] Mester L., Kovacs K., Racz B., Solti I., Atlasz T., Szabadfi K., Tamas A., Reglodi D. (2011). Pituitary Adenylate Cyclase-Activating Polypeptide Is Protective Against Oxidative Stress in Human Retinal Pigment Epithelial Cells. J. Mol. Neurosci..

[148] Bosnyak I., Farkas N., Molitor D., Meresz B., Patko E., Atlasz T., Vaczy A., Reglodi D. (2024). Optimization of an Ischemic Retinopathy Mouse Model and the Consequences of Hypoxia in a Time-Dependent Manner. Int. J. Mol. Sci..

[149] Ladea L., Zemba M., Calancea M.I., Călțaru M.V., Dragosloveanu C.D.M., Coroleucă R., Catrina E.L., Brezean I., Dinu V. (2024). Corneal Epithelial Changes in Diabetic Patients: A Review. Int. J. Mol. Sci..

[150] Yeung A., Dwarakanathan S. (2021). Diabetic Keratopathy. Dis. Mon..

[151] Hirabayashi T., Shibato J., Kimura A., Yamashita M., Takenoya F., Shioda S. (2022). Potential Therapeutic Role of Pituitary Adenylate Cyclase-Activating Polypeptide for Dry Eye Disease. Int. J. Mol. Sci..

[152] Maugeri G., D’Amico A.G., Castrogiovanni P., Saccone S., Federico C., Reibaldi M., Russo A., Bonfiglio V., Avitabile T., Longo A. (2019). PACAP through EGFR Transactivation Preserves Human Corneal Endothelial Integrity. J. Cell. Biochem..

[153] Maugeri G., D’Amico A.G., Amenta A., Saccone S., Federico C., Reibaldi M., Russo A., Bonfiglio V., Avitabile T., Longo A. (2020). Protective Effect of PACAP against Ultraviolet B Radiation-Induced Human Corneal Endothelial Cell Injury. Neuropeptides.

[154] Maugeri G., Longo A., D’Amico A.G., Rasà D.M., Reibaldi M., Russo A., Bonfiglio V., Avitabile T., D’Agata V. (2018). Trophic Effect of PACAP on Human Corneal Endothelium. Peptides.

[155] Wang Z.-Y., Alm P., Håkanson R. (1995). Distribution and Effects of Pituitary Adenylate Cyclase-Activating Peptide in the Rabbit Eye. Neuroscience.

[156] Fukiage C., Nakajima T., Takayama Y., Minagawa Y., Shearer T.R., Azuma M. (2007). PACAP Induces Neurite Outgrowth in Cultured Trigeminal Ganglion Cells and Recovery of Corneal Sensitivity after Flap Surgery in Rabbits. Am. J. Ophthalmol..

[157] Wu L., Wang J., Chen X., Hong A. (2015). Expression, Identification and Biological Effects of the Novel Recombination Protein, PACAP38-NtA, with High Bioactivity. Int. J. Mol. Med..

[158] Shioda S., Takenoya F., Hirabayashi T., Wada N., Seki T., Nonaka N., Nakamachi T. (2019). Effects of PACAP on Dry Eye Symptoms, and Possible Use for Therapeutic Application. J. Mol. Neurosci..

[159] Wang Z., Shan W., Li H., Feng J., Lu S., Ou B., Ma M., Ma Y. (2019). The PACAP-Derived Peptide MPAPO Facilitates Corneal Wound Healing by Promoting Corneal Epithelial Cell Proliferation and Trigeminal Ganglion Cell Axon Regeneration. Int. J. Biol. Sci..

[160] Ma Y., Zhao S., Wang X., Shen S., Ma M., Xu W., Hong A. (2015). A New Recombinant PACAP-Derived Peptide Efficiently Promotes Corneal Wound Repairing and Lacrimal Secretion. Invest. Ophthalmol. Vis. Sci..

[161] Delcourt N., Thouvenot E., Chanrion B., Galéotti N., Jouin P., Bockaert J., Marin P. (2007). PACAP Type I Receptor Transactivation Is Essential for IGF-1 Receptor Signalling and Antiapoptotic Activity in Neurons. EMBO J..

[162] Maugeri G., D’Amico A.G., Bucolo C., D’Agata V. (2019). Protective Effect of PACAP-38 on Retinal Pigmented Epithelium in an in Vitro and in Vivo Model of Diabetic Retinopathy through EGFR-Dependent Mechanism. Peptides.

[163] Maugeri G., D’Amico A.G., Saccone S., Federico C., Rasà D.M., Caltabiano R., Broggi G., Giunta S., Musumeci G., D’Agata V. (2021). Effect of PACAP on Hypoxia-Induced Angiogenesis and Epithelial–Mesenchymal Transition in Glioblastoma. Biomedicines.

[164] Moody T.W., Osefo N., Nuche-Berenguer B., Ridnour L., Wink D., Jensen R.T. (2012). Pituitary Adenylate Cyclase-Activating Polypeptide Causes Tyrosine Phosphorylation of the Epidermal Growth Factor Receptor in Lung Cancer Cells. J. Pharmacol. Exp. Ther..

[165] Hayden M.S., Ghosh S. (2008). Shared Principles in NF-KappaB Signaling. Cell.

[166] Chew S.M., Dua Avinashi S., Venkataraman K. (2025). Predictors of Incident Diabetic Peripheral Neuropathy: A Systematic Review of Longitudinal Studies in Patients with Diabetes Mellitus. Rev. Endocr. Metab. Disord..

[167] Zhu J., Hu Z., Luo Y., Liu Y., Luo W., Du X., Luo Z., Hu J., Peng S. (2024). Diabetic Peripheral Neuropathy: Pathogenetic Mechanisms and Treatment. Front. Endocrinol..

[168] Galiero R., Caturano A., Vetrano E., Beccia D., Brin C., Alfano M., Di Salvo J., Epifani R., Piacevole A., Tagliaferri G. (2023). Peripheral Neuropathy in Diabetes Mellitus: Pathogenetic Mechanisms and Diagnostic Options. Int. J. Mol. Sci..

[169] Tomczak J., Kapsa A., Boczek T. (2025). Adenylyl Cyclases as Therapeutic Targets in Neuroregeneration. Int. J. Mol. Sci..

[170] Shioda S., Nakamachi T. (2015). PACAP as a Neuroprotective Factor in Ischemic Neuronal Injuries. Peptides.

[171] Watanabe J., Seki T., Shioda S., Reglodi D., Tamas A. (2016). PACAP and Neural Development. Pituitary Adenylate Cyclase Activating Polypeptide—PACAP.

[172] Waschek J.A. (2002). Multiple Actions of Pituitary Adenylyl Cyclase Activating Peptide in Nervous System Development and Regeneration. Dev. Neurosci..

[173] Somogyvari-Vigh A., Reglodi D. (2004). Pituitary Adenylate Cyclase Activating Polypeptide: A Potential Neuroprotective Peptide. Curr. Pharm. Des..

[174] Pettersson L.M.E., Dahlin L.B., Danielsen N. (2004). Changes in Expression of PACAP in Rat Sensory Neurons in Response to Sciatic Nerve Compression. Eur. J. Neurosci..

[175] Woodley P.K., Min Q., Li Y., Mulvey N.F., Parkinson D.B., Dun X. (2019). Distinct VIP and PACAP Functions in the Distal Nerve Stump During Peripheral Nerve Regeneration. Front. Neurosci..

[176] Ogata K., Shintani N., Hayata-Takano A., Kamo T., Higashi S., Seiriki K., Momosaki H., Vaudry D., Vaudry H., Galas L. (2015). PACAP Enhances Axon Outgrowth in Cultured Hippocampal Neurons to a Comparable Extent as BDNF. PLoS ONE.

[177] Nakajima E., Walkup R.D., Fujii A., Shearer T.R., Azuma M. (2013). Pituitary Adenylate Cyclase-Activating Peptide Induces Neurite Outgrowth in Cultured Monkey Trigeminal Ganglion Cells: Involvement of Receptor PAC1. Mol. Vis..

[178] Guirland C., Buck K.B., Gibney J.A., DiCicco-Bloom E., Zheng J.Q. (2003). Direct CAMP Signaling through G-Protein-Coupled Receptors Mediates Growth Cone Attraction Induced by Pituitary Adenylate Cyclase-Activating Polypeptide. J. Mol. Neurosci..

[179] Pandey S., Mudgal J. (2022). A Review on the Role of Endogenous Neurotrophins and Schwann Cells in Axonal Regeneration. J. Neuroimmune Pharmacol..

[180] Armstrong B.D., Abad C., Chhith S., Cheung-Lau G., Hajji O.E., Nobuta H., Waschek J.A. (2008). Impaired Nerve Regeneration and Enhanced Neuroinflammatory Response in Mice Lacking Pituitary Adenylyl Cyclase Activating Peptide. Neuroscience.

[181] Maugeri G., D’Amico A.G., Musumeci G., Reglodi D., D’Agata V. (2020). Effects of PACAP on Schwann Cells: Focus on Nerve Injury. Int. J. Mol. Sci..

[182] Castorina A., Reglodi D., Tamas A. (2016). Multiple Actions of Pituitary Adenylate Cyclase-Activating Polypeptide (PACAP) in Schwann Cell Biology. Pituitary Adenylate Cyclase Activating Polypeptide—PACAP.

[183] Castorina A., Waschek J.A., Marzagalli R., Cardile V., Drago F. (2015). PACAP Interacts with PAC1 Receptors to Induce Tissue Plasminogen Activator (TPA) Expression and Activity in Schwann Cell-Like Cultures. PLoS ONE.

[184] Castorina A., Tiralongo A., Giunta S., Carnazza M.L., Rasi G., D’Agata V. (2008). PACAP and VIP Prevent Apoptosis in Schwannoma Cells. Brain Res..

[185] Castorina A., Scuderi S., D’Amico A.G., Drago F., D’Agata V. (2014). PACAP and VIP Increase the Expression of Myelin-Related Proteins in Rat Schwannoma Cells: Involvement of PAC1/VPAC2 Receptor-Mediated Activation of PI3K/Akt Signaling Pathways. Exp. Cell Res..

[186] Musumeci G., Leggio G., Marzagalli R., Al-Badri G., Drago F., Castorina A. (2018). Identification of Dysregulated MicroRNA Networks in Schwann Cell-Like Cultures Exposed to Immune Challenge: Potential Crosstalk with the Protective VIP/PACAP Neuropeptide System. Int. J. Mol. Sci..

[187] Chen Q., Zhang X.-Y., Wang Y.-P., Fu Y.-J., Cao F., Xu Y.-N., Kong J.-G., Tian N.-X., Xu Y., Wang Y. (2023). Unveiling Adcyap1 as a Protective Factor Linking Pain and Nerve Regeneration through Single-Cell RNA Sequencing of Rat Dorsal Root Ganglion Neurons. BMC Biol..

[188] Yamaguchi M., Noda-Asano S., Inoue R., Himeno T., Motegi M., Hayami T., Nakai-Shimoda H., Kono A., Sasajima S., Miura-Yura E. (2024). Dipeptidyl Peptidase (DPP)-4 Inhibitors and Pituitary Adenylate Cyclase-Activating Polypeptide, a DPP-4 Substrate, Extend Neurite Outgrowth of Mouse Dorsal Root Ganglia Neurons: A Promising Approach in Diabetic Polyneuropathy Treatment. Int. J. Mol. Sci..

[189] Baskozos G., Sandy-Hindmarch O., Clark A.J., Windsor K., Karlsson P., Weir G.A., McDermott L.A., Burchall J., Wiberg A., Furniss D. (2020). Molecular and Cellular Correlates of Human Nerve Regeneration: ADCYAP1/PACAP Enhance Nerve Outgrowth. Brain.

[190] Yevgi R., Laloğlu E., Bilge N. (2023). High Plasma Calcitonin Gene-Related Peptide and Serum Pituitary Adenylate Cyclase-Activating Polypeptide Levels in Patients with Neuropathic Pain. Rev Neurol.

[191] Khan M.-A., Batuman V., Reglodi D., Tamas A. (2016). Renoprotective Effects of Pituitary Adenylate Cyclase-Activating Polypeptide 38 (PACAP38). Pituitary Adenylate Cyclase Activating Polypeptide—PACAP.

[192] László E., Kiss P., Horváth G., Szakály P., Tamás A., Reglődi D. (2014). The Effects of Pituitary Adenylate Cyclase Activating Polypeptide in Renal Ischemia/Reperfusion. Acta Biol. Hung..

[193] Szakaly P., Laszlo E., Kovacs K., Racz B., Horvath G., Ferencz A., Lubics A., Kiss P., Tamas A., Brubel R. (2011). Mice Deficient in Pituitary Adenylate Cyclase Activating Polypeptide (PACAP) Show Increased Susceptibility to in Vivo Renal Ischemia/Reperfusion Injury. Neuropeptides.

[194] Sparks J., Jungling A., Kiss G., Hiripi L., Pham D., Tamas A., Hoffmann O., Bardosi S., Miseta A., Reglodi D. (2021). Presence of Systemic Amyloidosis in Mice with Partial Deficiency in Pituitary Adenylate Cyclase-Activating Polypeptide (PACAP) in Aging. Appl. Sci..

[195] Wang Y., Liu Y., Gu W., Cai B., Lei M., Luo Y., Zhang N. (2025). Association of Immune-Inflammation Indexes with Incidence and Prognosis of Diabetic Nephropathy: A Systematic Review and Meta-Analysis. Front. Endocrinol..

[196] Sakamoto K., Kuno K., Takemoto M., He P., Ishikawa T., Onishi S., Ishibashi R., Okabe E., Shoji M., Hattori A. (2015). Pituitary Adenylate Cyclase-Activating Polypeptide Protects Glomerular Podocytes from Inflammatory Injuries. J. Diabetes Res..

[197] Solymar M., Ivic I., Balasko M., Fulop B.D., Toth G., Tamas A., Reman G., Koller A., Reglodi D. (2018). Pituitary Adenylate Cyclase-Activating Polypeptide Ameliorates Vascular Dysfunction Induced by Hyperglycaemia. Diabetes Vasc. Dis. Res..

[198] Pulipaka S., Singuru G., Sahoo S., Shaikh A., Thennati R., Kotamraju S. (2023). Therapeutic Efficacies of Mitochondria-Targeted Esculetin and Metformin in the Improvement of Age-Associated Atherosclerosis via Regulating AMPK Activation. Geroscience.

[199] Rácz B., Gasz B., Borsiczky B., Gallyas F., Tamás A., Józsa R., Lubics A., Kiss P., Rőth E., Ferencz A. (2007). Protective Effects of Pituitary Adenylate Cyclase Activating Polypeptide in Endothelial Cells against Oxidative Stress-Induced Apoptosis. Gen. Comp. Endocrinol..

[200] Banki E., Sosnowska D., Tucsek Z., Gautam T., Toth P., Tarantini S., Tamas A., Helyes Z., Reglodi D., Sonntag W.E. (2015). Age-Related Decline of Autocrine Pituitary Adenylate Cyclase-Activating Polypeptide Impairs Angiogenic Capacity of Rat Cerebromicrovascular Endothelial Cells. J. Gerontol. Ser. A Biomed. Sci. Med. Sci..

[201] Baranoglu Kilinc Y., Dagistan Y., Kilinc E. (2025). Plasma Levels of Biomarkers Associated with Vasodilation and Neuroinflammation in Pediatric Patients with Head Trauma and Their Relationship with Clinical Characteristics of Patients. Child’s Nerv. Syst..

[202] Chang Q. (1997). [Experimental Study of the Effects of Pituitary Adenylate Cyclase-Activating Polypeptide (PACAP) and Its Mechanism on the Vascular Cell Components--the Possible Relationship between PACAP and Atherosclerosis]. Sheng Li Ke Xue Jin Zhan.

[203] Mey L., Bonaterra G.A., Hoffmann J., Schwarzbach H., Schwarz A., Eiden L.E., Weihe E., Kinscherf R. (2024). PAC1 Agonist Maxadilan Reduces Atherosclerotic Lesions in Hypercholesterolemic ApoE-Deficient Mice. Int. J. Mol. Sci..

[204] Rasbach E., Splitthoff P., Bonaterra G.A., Schwarz A., Mey L., Schwarzbach H., Eiden L.E., Weihe E., Kinscherf R. (2019). PACAP Deficiency Aggravates Atherosclerosis in ApoE Deficient Mice. Immunobiology.

[205] Castorina A., Giunta S., Mazzone V., Cardile V., D’Agata V. (2010). Effects of PACAP and VIP on Hyperglycemia-Induced Proliferation in Murine Microvascular Endothelial Cells. Peptides.

[206] Tian S., Wu T., Zhang Z., Lv S., Ji X., Zhao Z., Ma X., Wang J., Bi Y. (2025). Activation of Central Angiotensin-(1-7)/Mas Receptor Alleviates Synaptic Damage in Diabetes-Associated Cognitive Impairment via Modulating AKT/FOXO1/PACAP Axis. Int. J. Biol. Sci..

[207] Jansen M.I., Hrncir H., MacKenzie-Graham A., Waschek J.A., Brinkman J., Bradfield L.A., Withana M., Musumeci G., D’Agata V., Castorina A. (2025). Neuronal PAC1 Deletion Impairs Structural Plasticity. Life Sci..

[208] Wang Q., Wang Y., Li S., Shi J. (2023). PACAP–Sirtuin3 Alleviates Cognitive Impairment through Autophagy in Alzheimer’s Disease. Alzheimers Res. Ther..

[209] Heppner T.J., Hennig G.W., Nelson M.T., May V., Vizzard M.A. (2019). PACAP38-Mediated Bladder Afferent Nerve Activity Hyperexcitability and Ca2+ Activity in Urothelial Cells from Mice. J. Mol. Neurosci..

[210] Ojala J., Tooke K., Hsiang H., Girard B.M., May V., Vizzard M.A. (2019). PACAP/PAC1 Expression and Function in Micturition Pathways. J. Mol. Neurosci..

[211] Han X., Chen Y., Ha L., Yang J., Wang F., Chen H., Zhou Q., Long C., Qiu X., Chen Q. (2023). Effects of Electroacupuncture on Bladder Dysfunction and the Expression of PACAP38 in a Diabetic Rat Model. Front. Physiol..

[212] Shpakov A.O., Bondareva V.M., Chistiakova O. (2010). V [Functional State of Adenylyl Cyclase Signaling System in Reproductive Tissues of Rats with Experimental Type 1 Diabetes]. Tsitologiia.

[213] Shpakov A.O., Derkach K.V., Bondareva V.M. (2009). Changes in Hormone Sensitivity of the Adenylate Cyclase Signaling System in the Testicular Tissue of Rats with Neonatal Streptozotocin-Induced Diabetes. Bull. Exp. Biol. Med..

[214] Reed S., Taka E., Darling-Reed S., Soliman K.F.A. (2025). Neuroprotective Effects of Metformin Through the Modulation of Neuroinflammation and Oxidative Stress. Cells.

[215] Tao W., Yu Y., Tan D., Huang X., Huang J., Lin C., Yu R. (2025). Microbiota and Enteric Nervous System Crosstalk in Diabetic Gastroenteropathy: Bridging Mechanistic Insights to Microbiome-Based Therapies. Front. Cell. Infect. Microbiol..

[216] Palus K., Bulc M., Całka J., Zielonka Ł., Nowicki M. (2021). Diabetes Affects the Pituitary Adenylate Cyclase-Activating Polypeptide (PACAP)-Like Immunoreactive Enteric Neurons in the Porcine Digestive Tract. Int. J. Mol. Sci..

[217] Zhu L., Tamvakopoulos C., Xie D., Dragovic J., Shen X., Fenyk-Melody J.E., Schmidt K., Bagchi A., Griffin P.R., Thornberry N.A. (2003). The Role of Dipeptidyl Peptidase IV in the Cleavage of Glucagon Family Peptides. J. Biol. Chem..

[218] Lambeir A.-M., Durinx C., Proost P., Van Damme J., Scharpé S., De Meester I. (2001). Kinetic Study of the Processing by Dipeptidyl-peptidase IV/CD26 of Neuropeptides Involved in Pancreatic Insulin Secretion. FEBS Lett..

[219] Al-Badri G., Leggio G., Musumeci G., Marzagalli R., Drago F., Castorina A. (2018). Tackling Dipeptidyl Peptidase IV in Neurological Disorders. Neural Regen. Res..

[220] Bernstein H.-G., Keilhoff G., Dobrowolny H., Steiner J. (2023). The Many Facets of CD26/Dipeptidyl Peptidase 4 and Its Inhibitors in Disorders of the CNS—A Critical Overview. Rev. Neurosci..

[221] Nassar N.N., Al-Shorbagy M.Y., Arab H.H., Abdallah D.M. (2015). Saxagliptin: A Novel Antiparkinsonian Approach. Neuropharmacology.

[222] Ahrén B., Hughes T.E. (2005). Inhibition of Dipeptidyl Peptidase-4 Augments Insulin Secretion in Response to Exogenously Administered Glucagon-Like Peptide-1, Glucose-Dependent Insulinotropic Polypeptide, Pituitary Adenylate Cyclase-Activating Polypeptide, and Gastrin-Releasing Peptide in Mice. Endocrinology.

[223] Omar B., Ahrén B. (2014). Pleiotropic Mechanisms for the Glucose-Lowering Action of DPP-4 Inhibitors. Diabetes.

[224] Cheng Q., Cheng J., Cordato D., Gao J. (2020). Can Dipeptidyl Peptidase-4 Inhibitors Treat Cognitive Disorders?. Pharmacol. Ther..

[225] Agostini F., Masato A., Bubacco L., Bisaglia M. (2021). Metformin Repurposing for Parkinson Disease Therapy: Opportunities and Challenges. Int. J. Mol. Sci..

[226] DiGiovanni A., Shehaj A., Millar D., Tse C., Rizk E. (2025). Utility of Pharmacological Agents for Diabetes Mellitus in the Prevention of Alzheimer’s Disease: Comparison of Metformin, Glucagon-Like Peptide-1 (GLP-1) Agonists, Insulin, and Sulfonylureas. Cureus.

[227] Ullah A., Shen B. (2025). Immunomodulatory Effects of Anti-Diabetic Therapies: Cytokine and Chemokine Modulation by Metformin, Sodium-Glucose Cotransporter 2 Inhibitors, and Glucagon-like Peptide-1 Receptor Agonists (2013–2025). Eur. J. Med. Chem..

[228] Mandwie M., Karunia J., Niaz A., Keay K.A., Musumeci G., Rennie C., McGrath K., Al-Badri G., Castorina A. (2021). Metformin Treatment Attenuates Brain Inflammation and Rescues PACAP/VIP Neuropeptide Alterations in Mice Fed a High-Fat Diet. Int. J. Mol. Sci..

[229] Cherait A., Xifró X., Reglodi D., Vaudry D. (2025). More Than Three Decades After Discovery of the Neuroprotective Effect of PACAP, What Is Still Preventing Its Clinical Use?. J. Mol. Neurosci..

[230] Poujol de Molliens M., Létourneau M., Devost D., Hébert T.E., Fournier A., Chatenet D. (2018). New Insights about the Peculiar Role of the 28–38 C-Terminal Segment and Some Selected Residues in PACAP for Signaling and Neuroprotection. Biochem. Pharmacol..

[231] Hökfelt T., Bartfai T., Bloom F. (2003). Neuropeptides: Opportunities for Drug Discovery. Lancet Neurol..

[232] Meier J.J., Nauck M.A. (2015). Incretin-Based Therapies: Where Will We Be 50 Years from Now?. Diabetologia.

[233] Langoth N., Kalbe J., Bernkopschnurch A. (2005). Development of a Mucoadhesive and Permeation Enhancing Buccal Delivery System for PACAP (Pituitary Adenylate Cyclase-Activating Polypeptide). Int. J. Pharm..

[234] Reglodi D., Atlasz T., Jungling A., Szabo E., Kovari P., Manavalan S., Tamas A. (2018). Alternative Routes of Administration of the Neuroprotective Pituitary Adenylate Cyclase Activating Polypeptide. Curr. Pharm. Des..

[235] Xu W., Keith A.M., Ye W., Hu X., Southall N., Marugan J.J., Ferrer M., Henderson M.J., Sexton P.M., Deganutti G. (2025). Design of Peptide-Based PAC1 Antagonists Combining Molecular Dynamics Simulations and a Biologically Relevant Cell-Based Assay. Biochem. Pharmacol..

